# Retinoid-X-Receptors (α/β) in Melanocytes Modulate Innate Immune Responses and Differentially Regulate Cell Survival following UV Irradiation

**DOI:** 10.1371/journal.pgen.1004321

**Published:** 2014-05-08

**Authors:** Daniel J. Coleman, Gloria Garcia, Stephen Hyter, Hyo Sang Jang, Sharmeen Chagani, Xiaobo Liang, Lionel Larue, Gitali Ganguli-Indra, Arup K. Indra

**Affiliations:** 1 Department of Pharmaceutical Sciences, College of Pharmacy, Oregon State University, Corvallis, Oregon, United States of America; 2 Molecular and Cellular Biology Program, Oregon State University, Corvallis, Oregon, United States of America; 3 Developmental Genetics of Melanocytes, Institut Curie, Centre de Recherche, Orsay, France; CNRS UMR3347, Orsay, France; INSERM U1021, Orsay, France; 4 Dermatology Research Division, Oregon Health and Science University, Portland, Oregon, United States of America; University of Iceland, Iceland

## Abstract

Understanding the molecular mechanisms of ultraviolet (UV) induced melanoma formation is becoming crucial with more reported cases each year. Expression of type II nuclear receptor Retinoid-X-Receptor α (RXRα) is lost during melanoma progression in humans. Here, we observed that in mice with melanocyte-specific ablation of RXRα and RXRβ, melanocytes attract fewer IFN-γ secreting immune cells than in wild-type mice following acute UVR exposure, via altered expression of several chemoattractive and chemorepulsive chemokines/cytokines. Reduced IFN-γ in the microenvironment alters UVR-induced apoptosis, and due to this, the survival of surrounding dermal fibroblasts is significantly decreased in mice lacking RXRα/β. Interestingly, post-UVR survival of the melanocytes themselves is enhanced in the absence of RXRα/β. Loss of RXRs α/β specifically in the melanocytes results in an endogenous shift in homeostasis of pro- and anti-apoptotic genes in these cells and enhances their survival compared to the wild type melanocytes. Therefore, RXRs modulate post-UVR survival of dermal fibroblasts in a “non-cell autonomous” manner, underscoring their role in immune surveillance, while independently mediating post-UVR melanocyte survival in a “cell autonomous” manner. Our results emphasize a novel immunomodulatory role of melanocytes in controlling survival of neighboring cell types besides controlling their own, and identifies RXRs as potential targets for therapy against UV induced melanoma.

## Author Summary

Melanoma is the deadliest form of skin cancer. It derives from melanocytes, the melanin-producing cells of our skin, which give our skin its tone in addition to protecting it from harmful effects of ultraviolet radiation (UVR). Changes in the skin microenvironment, such as signaling from other cell types, can influence melanoma progression. While several key genes in melanoma development have been identified, the underlying mechanisms are complex; different combinations of mutations can result in melanoma formation and genetic profiles of tumors can vary greatly among patients. Therefore, identification of novel therapeutic targets is crucial. Our present study uses a tissue-specific gene ablation strategy to characterize a novel role of type II nuclear receptors [Retinoid-X-Receptors (RXRs)] in melanocytes to control UVR-induced skin immune responses and cell survival. Several of these observed changes are risk factors for melanoma progression and identify RXRs as potential drug targets for melanoma diagnosis, prevention, and treatment. This newly-discovered role of retinoid receptor signaling in immune surveillance can be studied in different types of cancer and in other diseases including metabolic syndromes and atherosclerosis. The identified pathway is ideal for targeting using specific ligands or small molecule modulators of RXR signaling in different cell types and tissues.

## Introduction

Malignant melanoma is the deadliest form of skin cancer. Understanding the molecular mechanisms behind melanoma formation is crucial for determining new pathways that can be utilized for therapeutic targeting. Retinoid-X-Receptors (RXRs) α, β, and γ (See Table 1 for all gene names, abbreviations, and IDs) are members of the nuclear hormone receptor (NR) superfamily, and act as central coordinators of cell signal transduction in many different tissue types through heterodimerization with several other NRs [1]. RXRs function as a ubiquitous DNA-binding transcription factor via ligand binding [1], [2] and heterodimerization with several other NRs [1], [3]. RXRs are able to interact with various transcriptional coactivators and/or corepressors [1]; adding an additional layer of complexity to their function. Thus, the role of RXRs in regulation of cellular processes is diverse and dynamic, and much is still unknown.

**Table 1:** IDs of all genes mentioned in text.

Gene Name	Gene Symbol	Species	Ensembl ID
Retinoid-X-Receptor α	*RXRα*	Human	ENSG00000186350
Retinoid-X-Receptor β	*RXRβ*	Human	ENSG00000204231
Retinoid-X-Receptor y	*RXRy*	Human	ENSG00000143171
Suppressor of Cytokine Signaling 1	*SOCS1*	Human	ENSG00000185338
Retinoid-X-Receptor α	*Rxrα*	Mouse	ENSMUSG00000015846
Retinoid-X-Receptor β	*Rxrβ*	Mouse	ENSMUSG00000039656
Retinoid-X-Receptor y	*Rxry*	Mouse	ENSMUSG00000015843
chemokine (C-C motif) receptor 2	*Ccr2*	Mouse	ENSMUSG00000049103
chemokine (C-C motif) ligand 2	*Ccl2*	Mouse	ENSMUSG00000035385
Chemokine (C-C motif) ligand 8	*Ccl8*	Mouse	ENSMUSG00000009185
EGF-like module containing, mucin-like, hormone receptor-like sequence 1	*Emr1* (common name F4/80)	Mouse	ENSMUSG00000004730
Interferon-y	*Ifng*	Mouse	ENSMUSG00000055170
Suppressor of Cytokine Signaling 1	*Socs1*	Mouse	ENSMUSG00000038037
Interferon gamma receptor 1	*Ifngr1*	Mouse	ENSMUSG00000020009
Integrin alpha M	*Itgam* (common name CD11B)	Mouse	ENSMUSG00000030786
CD8 antigen, beta chain 1	*Cd8b1* (common name CD8)	Mouse	ENSMUSG00000053044
Tyrosinase-related protein 1	*Tyrp1*	Mouse	ENSMUSG00000005994
Proliferating cell nuclear antigen	*Pcna*	Mouse	ENSMUSG00000027342
Chemokine (C-X-C motif) ligand 10	*Cxcl10*	Mouse	ENSMUSG00000034855
Chemokine (C-X-C motif) ligand 12	*Cxcl12*	Mouse	ENSMUSG00000061353
Slit homolog 2	*Slit2*	Mouse	ENSMUSG00000031558
Tumor necrosis factor	*Tnf*	Mouse	ENSMUSG00000024401
Chemokine (C-X-C motif) receptor 4	*Cxcr4*	Mouse	ENSMUSG00000045382
C-C motif chemokine 19	*Ccl19*	Mouse	ENSMUSG00000094661
Chemokine (C-X3-C motif) ligand 1	*Cx3Cl1*	Mouse	ENSMUSG00000031778
Chemokine (C-C motif) ligand 4	*Ccl4*	Mouse	ENSMUSG00000018930
Chemokine (C-C motif) receptor-like 2	*Ccrl2*	Mouse	ENSMUSG00000043953
Baculoviral IAP repeat-containing 5	*Birc5*	Mouse	ENSMUSG00000017716
B cell leukemia/lymphoma 2	*Bcl2*	Mouse	ENSMUSG00000057329
Fibroblast growth factor 1	*Fgf1*	Mouse	ENSMUSG00000036585
Neural cell adhesion molecule 1	*Ncam1*	Mouse	ENSMUSG00000039542
BCL2-associated agonist of cell death	*Bad*	Mouse	ENSMUSG00000024959
FBJ osteosarcoma oncogene	*Fos*	Mouse	ENSMUSG00000021250
Transformation related protein 53	*Trp53* (common name p53)	Mouse	ENSMUSG00000059552

In malignant human melanomas, loss of RXRα expression has been previously reported both in the melanoma cells themselves [4] and in the adjacent epidermal keratinocytes [5]. Also, epidermis-specific ablation of RXRα in a mouse model was shown to promote melanocyte proliferation after UV radiation (UVR) [6] and increased susceptibility to malignant melanomas after a multi-stage carcinogenesis treatment [5].

Chemokines are a family of small (8±14 kDa) polypeptide signaling molecules [7] that bind to transmembrane G protein-coupled receptors [7]. It has been previously reported that secretion of CCR2 ligands CCL2 and CCL8 from melanocytes following UVB radiation activates F4/80+ macrophages and results in their recruitment [8]. These macrophages infiltrate into the skin and secrete interferon-y (IFN-y); which mediates signaling that influences cell survival post-UVR [8]. Interferons have a complicated role in immunosurveillance with regard to cancer formation and progression. IFN-y secreted from macrophages recruited post-UVR have been shown to promote activation and survival of melanocytes and melanoma cells, suggesting a pro-tumorigenic role of IFN-y in skin [8]. In contrast, there is also evidence that IFN-y can act as an anti-tumorigenic agent [9]–[12].

In the present study, we discovered that specific ablation of RXRα and RXRβ in the melanocytes of the skin results in increased apoptosis of non-melanocytic cells in the dermis following UVR. However, the mutant melanocytes themselves exhibit slightly reduced apoptosis, suggesting an enhanced ability to survive UVR-induced DNA damage in the absence of RXRα/β. Interestingly, ablation of melanocytic RXRα and RXRβ results in decreased infiltration of immune cells such as F4/80+ macrophages, CD11B+ monocytes, CD8+ T-cells and mast cells into the dermal layer following UVR, as well as corresponding downregulated expression of IFN-γ, suggesting a defect in secretion of chemokines involved in immunomodulation. RT-qPCR analyses of UVR-exposed melanocytes revealed that loss of *Rxrα/β* results in significantly altered expression of both chemoattractive and chemorepulsive ligands implicated in chemotaxis of IFN-γ secreting immune cells. Treatment of primary melanocytes *in vitro* with either a small molecule agonist or antagonist of RXR function results in decreased UVR-induced apoptosis, suggesting that RXRs can mediate melanocyte apoptosis via multiple pathways utilizing both its activating and repressive functions. RT-qPCR analyses also revealed several post-UVR changes in expression of pro- and anti-apoptotic genes in melanocytes lacking *Rxrα/β* expression.

Our results suggest that ablation of RXRs α/β results in aberrant expression of several chemokines following UVR, resulting in decreased infiltration of macrophages and other immune cells that secrete IFN-γ. The corresponding decrease in IFN-γ may influence changes in survival of melanocytes, fibroblasts, and other cell types in the skin. It has been found previously that UVR-induced apoptosis in skin can stimulate clonal expansion of tumorigenic cells as the death of surrounding cells allows them room to expand [13]. As RXRα/β ablation exists only in the melanocytes, there is a cell-autonomous shift in homeostasis of pro- and anti-apoptotic gene expression in this cell type, which results in an enhanced survival of these cells post-UVR.

## Results

### Selective ablation of RXRα and RXRβ in melanocytes of *Tyr-Cre^tg/0^ Rxrα/β^L2/L2^* mice

**Figure 1:**
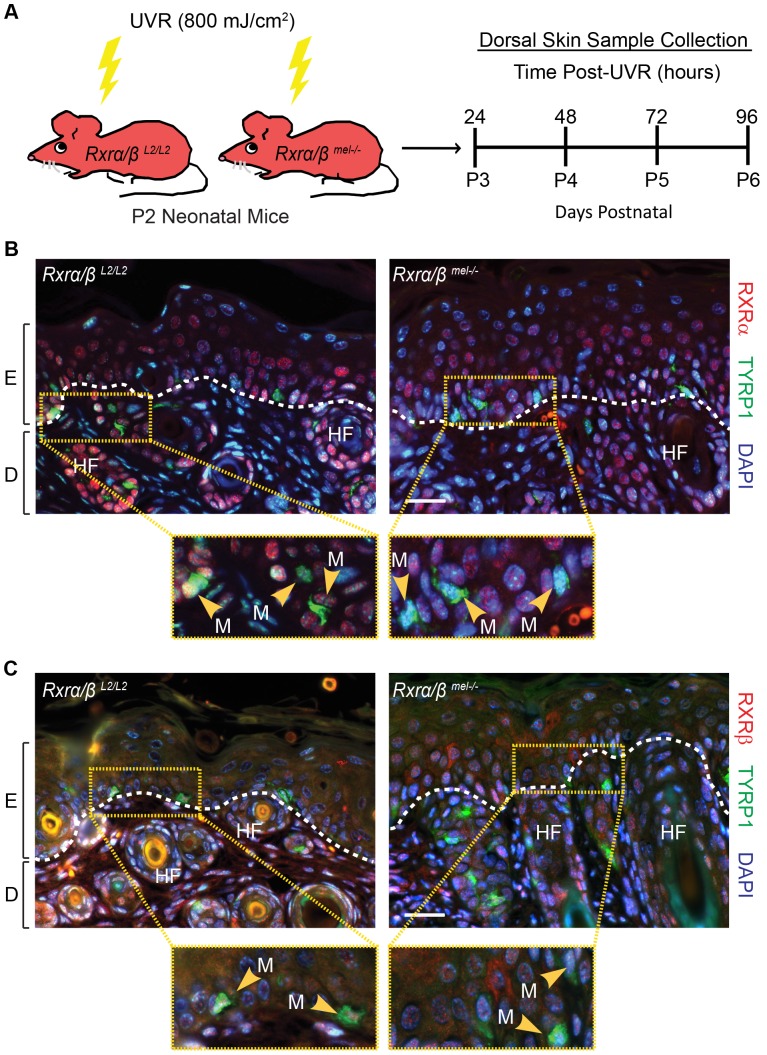
Loss of RXRs α and β are restricted to melanocytes in *Rxrα/β^mel−/−^* mice. (A) Experimental scheme for *in vivo* experiments. Floxed *Rxrα/β^L2/L2^* (controls) and *Rxrα/β^mel−/−^* mice are irradiated with UVR at Day 2 post-natal (P2) and samples collected at the indicated time points. (B, C) IHC for (B) RXRα or (C) RXRβ (red) co-labeled with melanocyte-specific marker TYRP1 (green) on skin sections collected from mice 96 hours post-UVR. Melanocytes are indicated by yellow arrows. Floxed *Rxrα/β^L2/L2^* control mice show melanocytes labeled for the RXRs, *Rxrα/β^mel−/−^* mice show melanocytes that are negative, as it is excised specifically in that cell type. E = epidermis, D = dermis, HF = hair follicle, M = melanocyte. Scale bar = 50 µm.

In order to selectively ablate RXRα and RXRβ in melanocytes, we bred mice carrying LoxP site-containing (floxed) *Rxrα* and *Rxrβ* alleles (*Rxrα/β^L2/L2^*) with hemizygous *Tyr-Cre* transgenic mice [14]. Macroscopically, *Tyr-Cre^tg/0^|Rxrα/β^L2/L2^* mice were indistinguishable from the control *Rxrα/β^L2/L2^* floxed mice (Figure S1A) and no effects on their viability were observed. In order to verify that RXRα and RXRβ were specifically deleted in melanocytes of *Tyr-Cre^tg/0^|Rxrα/β^L2/L2^* mice, we used immunohistochemistry (IHC) to examine skin sections from neonatal control and mutant mice collected after exposure to UVR (800 mJ/cm^2^ UVB) (Figure 1A), which dramatically increases numbers of extrafollicular melanocytes (Figure S1 D, E). Tissue sections were co-labeled with antibodies for tyrosinase-related protein 1 (TYRP1), which is localized specifically in the cytoplasm of melanocytes [15], [16] (Figure 1 B, C), and either RXRα (Figure 1B) or RXRβ (Figure 1C). We observed that in absence of the Cre transgene, cells positive for TYRP1 also show nuclear labeling for both RXRα (Figure 1B) and RXRβ (Figure 1C) in *Rxrα/β^L2/L2^* floxed mice. In presence of the Cre transgene, none of the TYRP1-labeled melanocytes showed positive staining for either receptor in the *Tyr-Cre^tg/0^|Rxrα/β^L2/L2^* mice, while all other cell types of the skin were positive for RXRα (Figure 1B) and RXRβ (Figure 1C), thereby confirming the generation of the *Rxrα/β^mel−/−^* mice. Altogether, our results indicate that *Rxrα/β^mel−/−^* mice constitute a model of selective ablation of both RXRα and RXRβ in the melanocytes of neonatal murine skin.

### Melanocytic ablation of RXRα and RXRβ differentially alters UVR-induced apoptosis in melanocytes and dermal cells of the skin

Histological and IHC characterization of *Rxrα/β^L2/L2^* (control, CT) and *Rxrα/β^mel−/−^* (mutant, MT) skin was performed on biopsies collected from neonatal mice at different time points following a single dose of UVR (Figure 1A). Measurement of epidermal thickness on Hematoxylin and Eosin (H&E) stained skin sections did not reveal any significant difference between CT and MT skin (Figure S1 B, C). Also, melanocyte numbers (Figure S1 D, E) and levels of UVR-induced DNA damage in melanocytes (Figure S2) were similar between the two groups.

**Figure 2:**
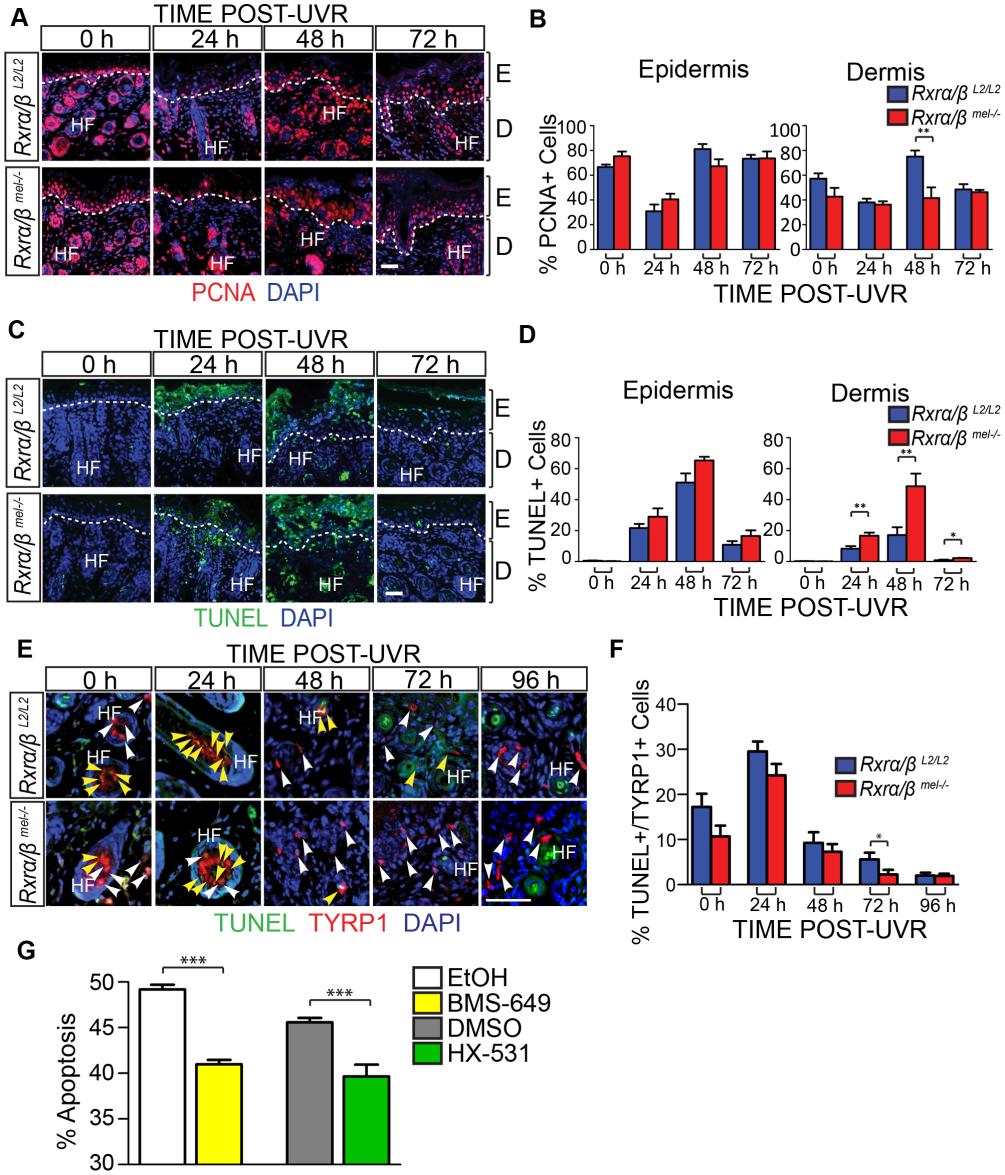
Loss of melanocytic RXRs α and β results in contrasting changes in cell survival in dermal skin cells and melanocytes following UV radiation. (A–F) Histological analyses of skin sections following a single dose of UVR. (A, B) IHC for the proliferation marker PCNA. Proliferating cells are indicated by red staining. ** = p≤0.01. (C, D) TUNEL assay to label apoptotic cells. Apoptotic cells are indicated by green staining. * = p≤0.05, ** = p≤0.01. (E, F) Hybrid TUNEL-IHC to analyze apoptosis specifically in melanocytes. Apoptotic cells are indicated by green staining, cells positive for the melanocyte-specific marker TYRP1 are indicated by red staining. White arrows indicate normal melanocytes, yellow arrows indicate apoptotic melanocytes. * = p≤0.1. (G) Apoptosis of cultured melanocytes 24 h post-UVR as a percentage of total cells as measured using Annexin V/Propidium Iodide staining. Cells were treated pre- and post-UVR with RXR agonist BMS-649, antagonist HX-531, or their vehicles as controls (EtOH and DMSO, respectively). *** = p≤0.001. For all images: DAPI (blue) was used to counterstain nuclei. E = epidermis, D = dermis, HF = hair follicle. Scale bars = 50 µm.

IHC analysis for proliferation marker PCNA revealed a significant reduction in proliferation of cells in the dermal layer that was most prominent at 48 hours post-UVR (Figure 2 A, B). Interestingly, we also observed a marked increase in apoptosis of the dermal cells in the MT skin compared to the CT skin using a TUNEL assay (Figure 2 C, D). This difference was significant at 24 and 48 hours post-UVR (Figure 2 C, D).

We next employed a hybrid TUNEL-IHC assay (with anti-TYRP1 antibody) to specifically evaluate melanocyte apoptosis (Figure 2E). In contrast to the overall dermal apoptosis, we observed a trend in reduced percentage of apoptotic melanocytes in MT skin compared to the CT skin at each time point post-UVR. The reduction in percent apoptotic melanocytes was significant at 72 hours post-UVR (Figure 2 E, F). To corroborate this result we utilized an *in vitro* assay using cultured primary murine melanocytes. Growth medium was supplemented with either a small-molecule agonist (BMS-649) or antagonist (HX-531) of RXRs in order to mimic loss of RXRα/β functions; by simulating relief of transcriptional repression or loss of transcriptional activation functions with addition of an agonist or antagonist, respectively. The treated primary melanocytes were then subjected to a single dose of UVR and given fresh medium supplemented with either agonist or antagonist. 24 hours post-UVR, apoptosis of the melanocytes was quantified using an Annexin V/Propidium Iodide cytometry assay. Compared to vehicle controls, apoptosis of the primary melanocytes was reduced when growth medium was supplemented with either an RXR agonist or antagonist (Figure 2G). The above results suggest a non-cell autonomous role in modulating survival of dermal cells and a cell autonomous role of melanocytic RXRα/β in mediating melanocyte homeostasis post-UVR, possibly via distinct mechanisms.

### Altered inflammatory responses in the skin of *Rxrα/β^mel−/−^* mice following exposure to UV radiation

**Figure 3:**
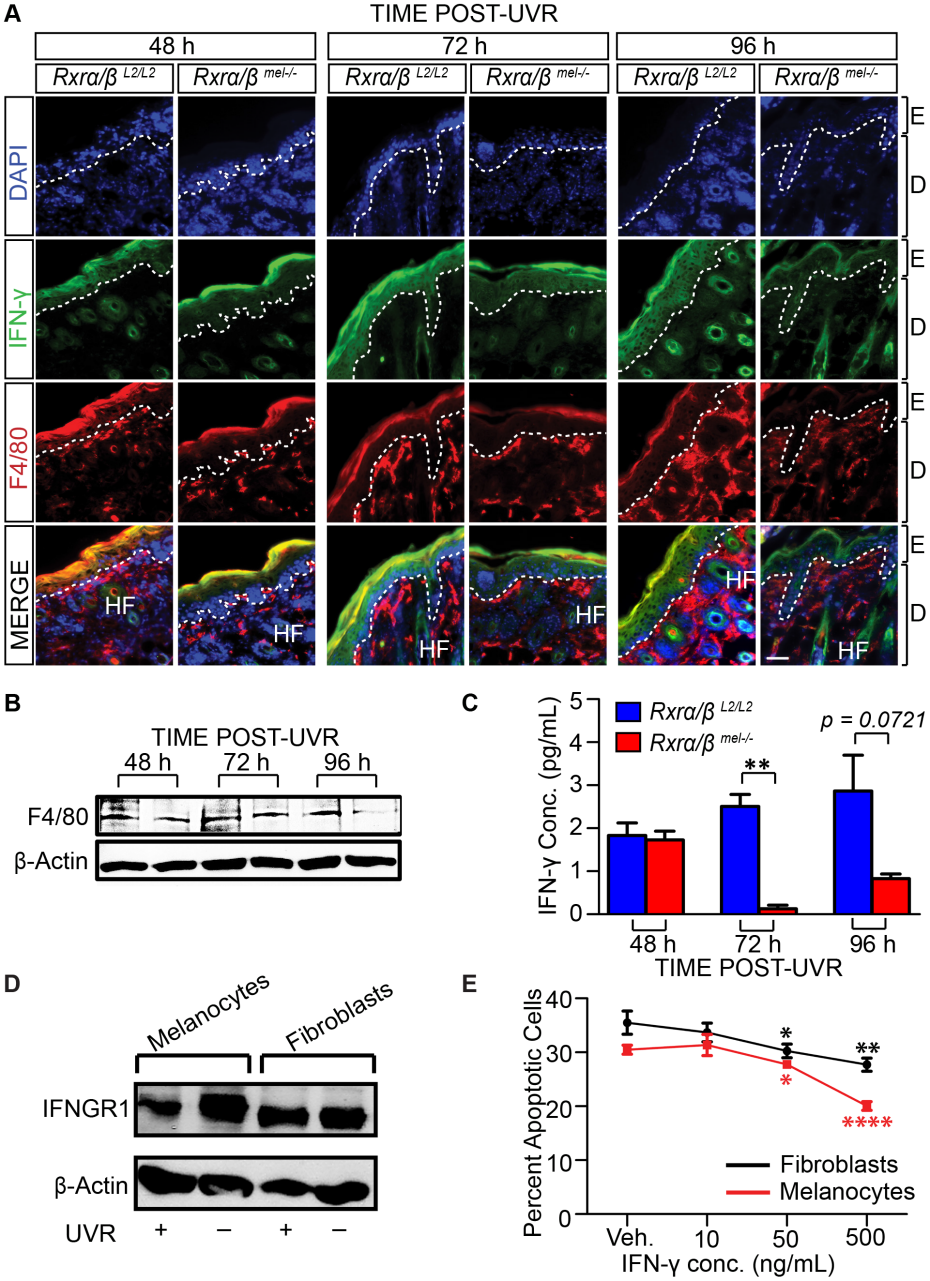
Loss of melanocytic RXRs α and β results in reduced monocyte/macrophage infiltration and corresponding reduced interferon-γ (IFN- γ) expression following UV radiation. IFN-γ influences cell survival post-UVR *in vitro*. (A) IHC using antibodies specific for IFN- γ (green) and macrophage antigen F4/80 (red) on skin sections collected from mice post-UVR. DAPI (blue) was used to counterstain nuclei. E = epidermis, D = dermis, HF = hair follicle. Scale bars = 50 µm. (B) Western Blot for macrophage antigen F4/80 in skin lysates collected post-UVR. (C) ELISA assay for IFN- γ in skin lysates collected post-UVR. ** = p≤0.01. (D, E) IFN- γ acts as a positive effector of post-UV survival in both melanocytes and fibroblasts. (D) Western blot of Interferon-γ receptor 1 (INFGR1) expression in cultured primary melanocytes and fibroblasts pre- and 24 h post-UVR. (E) Apoptosis of cultured fibroblasts and melanocytes 24 h post-UVR as a percentage of total cells as measured using Annexin V/Propidium Iodide. Immediately after UV treatment, culture medium was replaced with fresh medium containing several concentrations of recombinant IFN- γ. * = p≤0.05, ** = p≤0.01, **** = p≤0.0001.

It has been reported that in response to UVR, melanocytes secrete chemokines that mediate infiltration of macrophages [8], which further secrete interferon-γ (IFN-γ) that influences melanocyte survival [8]. We hypothesized that IFN-γ can also influence survival of other cells post-UVR; and the changes we observed in levels of apoptosis in the skin of *Rxrα/β^mel−/−^* mice may be due to altered immune responses. To test that, we first performed two-color IHC for IFN-γ expression and presence of macrophages using anti-IFN-γ and anti-F4/80 (a transmembrane cell surface protein associated with macrophages [17]) antibodies on CT and MT skin at different time points post-UVR. In comparison to control mice, MT skin exhibited moderately decreased IFN-γ expression and reduced F4/80+ labeling at 48 and 72 hours post-UVR, and a marked reduction of staining for both IFN-γ and F4/80+ cells at 96 hours post-UVR (Figure 3A). Western blot for F4/80, using a different antibody than used for IHC (Table 2), also revealed decreased F4/80 expression in skin lysates from MT skin compared to controls (Figure 3B). An ELISA assay confirmed a significant decrease in IFN-γ level in skin lysates from *Rxrα/β^mel−/−^* mice at 72 and 96 h post-UVR compared to controls (Figure 3C). We also observed reductions in CD11B+ monocytes, CD8+ T-cells, and mast cells (Figure S3 A–C). It is of note that F4/80 expression was previously reported to be required for CD8+ T-cell activation [18]. These data indicate that ablation of melanocytic RXRα and RXRβ results in reduced immune cell recruitment, which could result in reduced secretion of IFN-γ after exposure to UV radiation.

**Table 2:** List of antibodies used during experimental procedures.

Antibody	Host	Source	Application	Dilution/Conc.
anti-PEP1 (TYRP1)	Rabbit	NIH (kindly provided by V. Hearing)	IHC-P	1∶1000
anti-PCNA	Mouse	Abcam (ab29)	IHC-P	1∶6000
anti-RXRα	Mouse	Millipore (MAB5478)	IHC-P	1∶1000
anti-RXRα	Rabbit	Santa Cruz (sc553)	WB, ChIP	1∶250, 2.0 µg/ChIP
anti-RXRβ	Mouse	Abcam (ab2815)	IHC-P	1∶500
anti-F4/80	Rabbit	Abcam (ab74383)	WB	1∶750
anti-F4/80	Rat	BioLegend (122602)	IHC-Fr	1∶200
anti-IFN-γ	Rabbit	LSBio (C85867)	IHC-Fr	1∶200
anti-CPD	Mouse	(KB: MC-062)	IHC-P	1∶1000
anti-8-OHdG	Mouse	JaICA (MOG-020P)	IHC-P	7.5 µg/mL
anti-CD3	Rabbit	Abcam (ab16669)	IHC-P	1∶1000
anti-CD8	Rat	Abcam (ab22378)	IHC-Fr	1∶100
anti-CD11B	Rat	Abcam (ab8878)	IHC-Fr	1∶50
anti-IFN-γ R1	Armenian Hamster	R&D Systems (MAB10261)	WB	1.0 µg/mL
normal Rabbit IgG	Rabbit	Santa Cruz (sc2027)	ChIP	2.0 µg/ChIP
FITC-anti-CD45	Rat	BD Pharmingen (553079)	FACS	0.2 µg/10^6^ cells
PE-anti-CD117	Mouse	eBioscience (12-1172-81)	FACS	0.2 µg/10^6^ cells

***IHC-P = Immunohistochemistry - Paraffin Sections,***

***IHC-Fr = Immunohistochemistry - Frozen Sections, WB = Western Blot,***

***ChIP = Chromatin IP FACS = Fluorescence-activated cell sorting.***

As a complementary experiment, we wanted to determine how IFN-γ affects the survival of fibroblasts and melanocytes post-UVR. To that end, we irradiated both fibroblasts and melanocytes *in vitro* and examined the effects 24 h post-UVR. First, we confirmed that both cell types express the primary receptor for IFN-γ (IFNGR1) pre- and 24 h post-UVR using Western Blot (Figure 3D). We then analyzed apoptosis in cells cultured with recombinant murine IFN-γ post-UVR, using Annexin V/Propidium Iodide Staining. We observed that increasing concentration of IFN-γ resulted in a corresponding dose-dependent reduction in apoptosis levels of both fibroblasts and melanocytes following exposure to UVR (Figure 3E). These results suggest that secretion of IFN-γ in the microenvironment, which is reduced in *Rxrα/β^mel−/−^* mice compared to controls, is a key factor for post-UVR cell survival.

### Knockdown of RXRα and RXRβ in primary melanocytes results in alteration of the secretory chemokine/cytokine profile, as well as expression of genes involved in apoptosis

**Figure 4:**
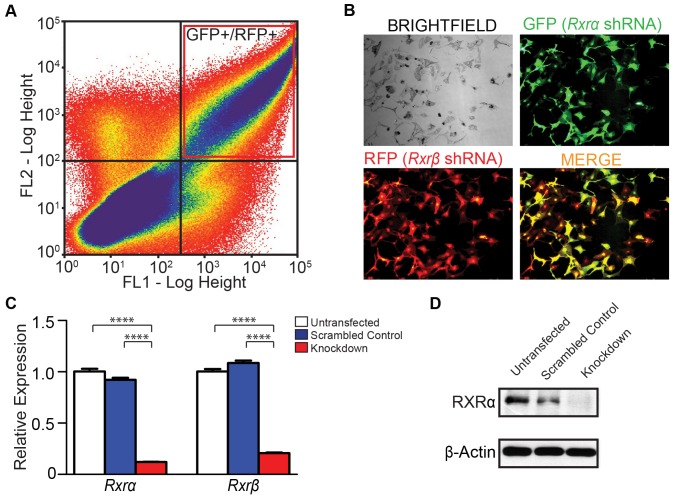
Generation of *Rxrα/Rxrβ* double knockdown murine melanocytes. (A) Primary murine melanocytes were transfected using a mixture of two shRNA-expressing plasmids targeting either *Rxrα* or *Rxrβ*. The *Rxrα* shRNA plasmid contains a GFP marker gene; *Rxrβ* shRNA contains a RFP marker gene. Cells positive for both GFP and RFP were sorted using FACS and re-cultured. (B) Re-cultured melanocytes following FACS express both GFP and RFP, indicating expression of both *Rxrα* and *Rxrβ* shRNA constructs. (C) RT-qPCR for measurement of *Rxrα/β* mRNA expression **** = p≤0.0001. (D) Western Blot for protein expression of RXRα in FACS-sorted cells.

We hypothesized that loss of RXRα/β may result in aberrant expression of chemokine ligands and/or receptors that are involved in immune cell infiltration; which in turn may influence survival of other skin cell types, particularly those in the dermis. We also examined the effects of RXRα/β loss on apoptosis-related genes; as mimicking functional RXR loss using an agonist or antagonist reduces post-UVR apoptosis of melanocytes *in vitro* (Figure 2G). To that end, we employed an RNAi-based knockdown strategy of both RXRα and RXRβ in primary murine melanocytes (Figure 4, see Materials and Methods for details). We used plasmids expressing shRNA constructs specifically targeting either *Rxrα* or *Rxrβ* transcripts (See Table 3 for details). The plasmid expressing *Rxrα*-targeting shRNA contains a GFP marker gene, while the *Rxrβ*-targeting shRNA plasmid encodes an RFP marker. The two different fluorescent marker genes allowed us to co-transfect both constructs into primary murine melanocytes, isolate the cell population expressing both plasmids by Fluorescence-Activated Cell Sorting (FACS) (Figure 4A) and then re-culture the isolated double positive cells (Figure 4B). We verified that knockdown was successful at both the RNA level using RT-qPCR (Figure 4C) and at the protein level using Western blot (Figure 4D). We observed a compensatory upregulation of *Rxrβ* expression in cells in which only *Rxrα* was knocked down, but not vice-versa (Figure S4).

**Table 3:** 29mer shRNA constructs used for gene knockdown studies.

*Gene*	29mer shRNA Construct (5′-3′)
***Rxrα***	TGCCTGTAGAGAAGATTCTGGAAGCCGAG
***Rxrβ***	TTGCTGTGGAGCAGAAGAGTGACCAAGGC
***Scrambled Control***	GCACTACCAGAGCTAACTCAGATAGTACT

**Figure 5:**
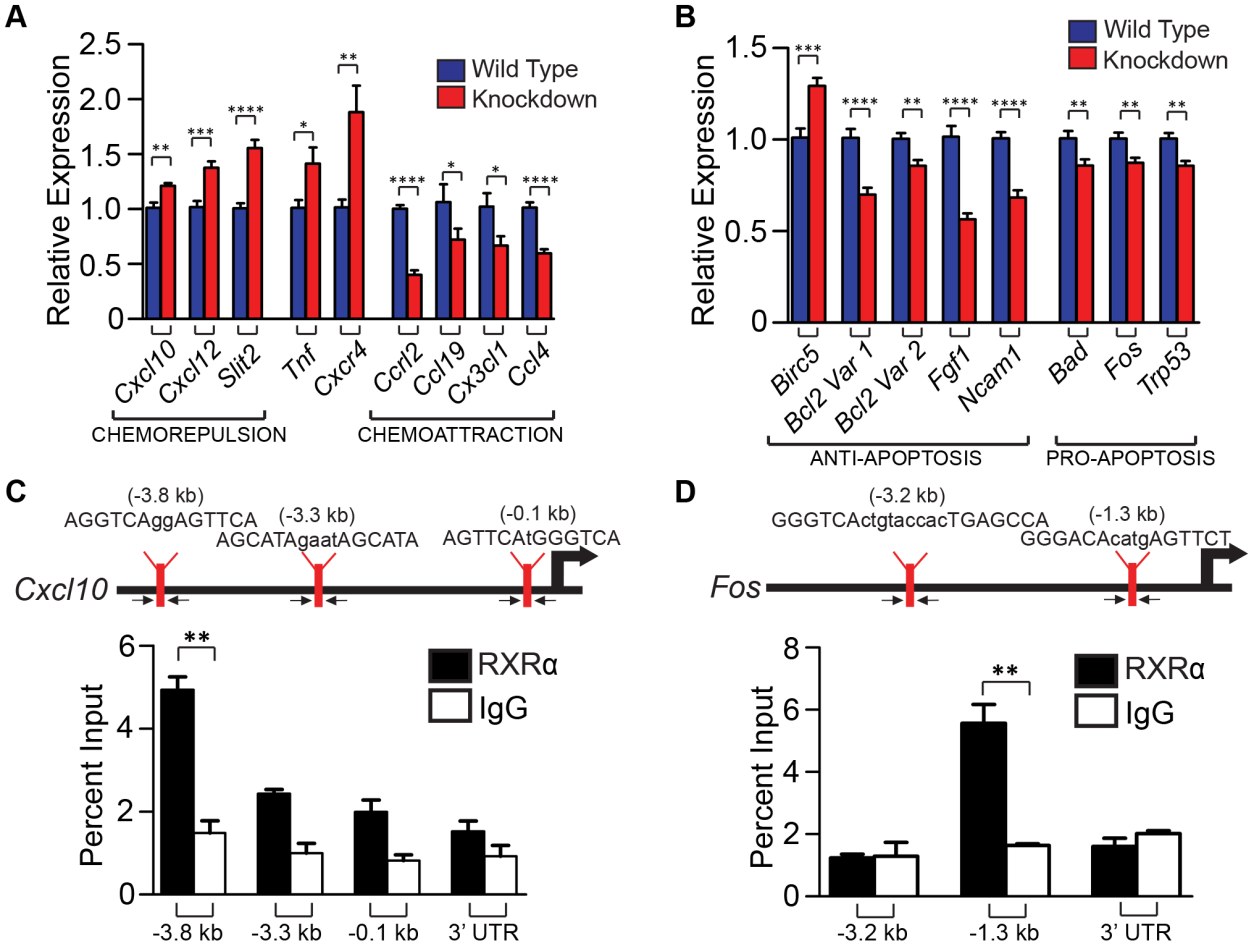
Ablation of RXR α and β in melanocytes results in altered expression of several chemokines post-UVR, as well as pro- and anti- apoptotic genes, some of which may be direct binding targets of RXRα. (A, B) Expression of genes found changed in RXRα/β double knockdown melanocytes post-UVR via RT-qPCR arrays (Figure S6). Expression of several chemokines (A) and apoptosis-related genes (B) were then re-verified by RT-qPCR using new primer sets. Primers spanning exon junctions were designed independently, and assays were performed on biological replicates of the sample used in the array. * = p≤0.05 **,  = p≤0.01, *** = p≤0.001, **** = p≤0.0001. (C, D) *In silico* analysis was used to find potential RXR response elements using Fuzznuc motif finder. These candidate binding sites were verified for enrichment in primary melanocytes using ChIP-RT-qPCR. A mock ChIP using a control IgG antibody was also performed. Arrows indicate targeting regions for primers. Genes negative for enrichment can be found in Figure S8. ** = p≤0.01.

The *Rxrα/β* double knockdown primary melanocytes were subjected to UVR (10 mJ/cm^2^ UV-B) and RNA isolated 6 hours later. We chose the UVR dose and time interval based on peak mRNA expression of chemokines *Ccl2* and *Ccl8* (Figure S5), two ligands secreted by melanocytes that are reported to influence macrophage infiltration in response to UVR [8]. cDNA synthesized from the RNA template was then applied to RT-qPCR arrays. We employed arrays for mouse chemokines/receptors (SA Biosciences, PAMM-022) and for mouse cancer/apoptosis (PAMM-033). Several mouse chemokines/receptors, as well as genes implicated in apoptosis, were found to be differentially up- or down-regulated compared to wild-type melanocytes (Figure S6 A–D; Figure 5 A, B). Expression of several candidate genes, which could contribute to reduced immune cell infiltration and reduced IFN-γ expression, were re-validated in new biological replicates by RT-qPCR using our own primer sets (for details see Table 4). In particular, expressions of *Cxcl10*, *Cxcl12*, *Slit2*, *Tnf* and *Cxcr4* were confirmed to be upregulated as determined by the array, while *Ccl19, Cx3Cl1, Ccl4, and Cclr2* were confirmed to be downregulated (Figure 5A). Additionally, several candidate genes which may contribute to the enhanced survival in the melanocytes themselves were found to be dysregulated. In particular, anti-apoptotic gene *Birc5* was found to be upregulated, while anti-apoptotic genes *Bcl2*, *Fgf1*, and *Ncam1* were downregulated (Figure 5B). Pro-apoptotic genes *Bad*, *Fos*, and *Trp53* were also downregulated (Figure 5B). In order to confirm that these genes were also altered in the mouse model, we performed FACS to isolate CD117+/CD45− melanocytes from mouse skin [19] 96 h post-UVR (Figure S7 A,B). Similar trends in dysregulated mRNA expression was observed for several genes in cells isolated from *Rxrα/β^mel−/−^* mice compared to *Rxrα/β* double knockdown cells; specifically chemokines/receptors *Cxcl10*, *Slit2*, *Ccrl2*, *Ccl19*, *Cx3Cl1*, and *Ccl4* (Figure S7C) and apoptosis-related genes *Fgf1*, *Bad*, and *Trp53* (Figure S7D). It is of note that *Slit2* had no detectable expression in cells collected from control mice; expression was only seen in *Rxrα/β^mel−/−^* cells (Figure S7C). Altogether, results suggest that melanocytic RXRα and RXRβ regulate expression of genes encoding chemokines and cytokines involved in chemoattraction/chemorepulsion of immune cells such as macrophage; as well as genes involved in melanocyte apoptosis/survival.

**Table 4:** List of primer sets used for RT-qPCR analysis of mRNA expression.

*Gene*	Ensembl ID (mouse)	Forward Primer (5′-3′)	Reverse Primer (5′-3′)
***Atm***	ENSMUSG00000034218	GATCTGCTCATTTGCTGCCG	GTGTGGTGGCTGATACATTTGAT
***Bad***	ENSMUSG00000024959	AAGTCCGATCCCGGAATCC	GCTCACTCGGCTCAAACTCT
***Bcl2 Var. 1***	ENSMUSG00000057329	ATGCCTTTGTGGAACTATATGGC	GGTATGCACCCAGAGTGATGC
***Bcl2 Var. 2***	ENSMUSG00000057329	GCTACCGTCGTGACTTCGC	CCCCACCGAACTCAAAGAAGG
***Birc5***	ENSMUSG00000017716	GAGGCTGGCTTCATCCACTG	CTTTTTGCTTGTTGTTGGTCTCC
***Ccl19***	ENSMUSG00000094661	AGACTGCTGCCTGTCTGTGA	TCTTCAGTCTTCGGATGATGC
***Ccl2***	ENSMUSG00000035385	TAAAAACCTGGATCGGAACCAAA	GCATTAGCTTCAGATTTACGGGT
***Ccl4***	ENSMUSG00000018930	TTCCTGCTGTTTCTCTTACACCT	CTGTCTGCCTCTTTTGGTCAG
***Ccl7***	ENSMUSG00000035373	GCTGCTTTCAGCATCCAAGTG	CCAGGGACACCGACTACTG
***Ccl8***	ENSMUSG00000009185	CTGGGCCAGATAAGGCTCC	CATGGGGCACTGGATATTGTT
***Ccrl2***	ENSMUSG00000043953	GCCCCGGACGATGAATATGAT	CACCAAGATAAACACCGCCAG
***Cdk2***	ENSMUSG00000025358	ATGGAGAACTTCCAAAAGGTGG	CAGTCTCAGTGTCGAGCCG
***Cmtm4***	ENSMUSG00000096188	CAAGGTCGCCCAAGTGATTCT	GATTCCAGTTGATCTGGGGGA
***Cmtm6***	ENSMUSG00000032434	ATGGAGAACGGAGCGGTCTA	CACACTCGGACACAACCTCT
***Cx3Cl1***	ENSMUSG00000031778	CGCGTTCTTCCATTTGTGTA	TAGCTGATAGCGGATGAGCA
***Cxcl10***	ENSMUSG00000034855	CCAAGTGCTGCCGTCATTTTC	TCCCTATGGCCCTCATTCTCA
***Cxcl12***	ENSMUSG00000061353	TGCATCAGTGACGGTAAACCA	CACAGTTTGGAGTGTTGAGGAT
***Cxcl5***	ENSMUSG00000029371	ATCCCCAGCGGTTCCATCT	GCGGCTATGACTGAGGAAGG
***Cxcr4***	ENSMUSG00000045382	GACTGGCATAGTCGGCAATG	AGAAGGGGAGTGTGATGACAAA
***Cxcr5***	ENSMUSG00000047880	ATGAACTACCCACTAACCCTGG	TGTAGGGGAATCTCCGTGCT
***Cxcr7***	ENSMUSG00000044337	GCAAGAGATGGCCAAGAGAC	GTGTCCACCACAATGCAGTC
***Fgf1***	ENSMUSG00000036585	CAGCTCAGTGCGGAAAGTG	TGTCTGCGAGCCGTATAAAAG
***Fos***	ENSMUSG00000021250	CGGGTTTCAACGCCGACTA	TTGGCACTAGAGACGGACAGA
***Itga2***	ENSMUSG00000015533	GGGGACCGGAGGCTTTCTA	GGCCTGTCACAAACTTTACCAAA
***Itga3***	ENSMUSG00000001507	CCTCTTCGGCTACTCGGTC	CCAGTCCGGTTGGTATAGTCATC
***Myc***	ENSMUSG00000022346	ATGCCCCTCAACGTGAACTTC	CGCAACATAGGATGGAGAGCA
***Ncam1***	ENSMUSG00000039542	GGGGAGGATGCTGTGATTGTC	GCGGTAAGTACCCTCATCTGT
***Pdgfb***	ENSMUSG00000000489	CATCCGCTCCTTTGATGATCTT	GTGCTCGGGTCATGTTCAAGT
***Pik3r1***	ENSMUSG00000041417	GCAGAGGGCTACCAGTACAGA	CTGAATCCAAGTGCCACTAAGG
***Pten***	ENSMUSG00000013663	TGGATTCGACTTAGACTTGACCT	GCGGTGTCATAATGTCTCTCAG
***Rb1***	ENSMUSG00000022105	TTGGAGTCCGATTGTATTACCGT	AGCACAGGCCAGTAAAGACAT
***Rxrα***	ENSMUSG00000015846	GATATCAAGCCGCCACTAGG	TGTTGTCTCGGCAGGTGTAG
***Rxrβ***	ENSMUSG00000039656	CACCTCTTACCCCTTCAGCA	GAGCGACACTGTGGAGTTGA
***Slit2***	ENSMUSG00000031558	GGCAGACACTGTCCCTATCG	ATCTATCTTCGTGATCCTCGTGA
***Thbs2***	ENSMUSG00000031558	CTGGGCATAGGGCCAAGAG	GTCTTCCGGTTAATGTTGCTGAT
***Tnf***	ENSMUSG00000024401	CCTGTAGCCCACGTCGTAG	GGGAGTAGACAAGGTACAACCC
***Trp53***	ENSMUSG00000059552	CACAGCACATGACGGAGGTC	TCCTTCCACCCGGATAAGATG
***Tyr***	ENSMUSG00000004651	TTACTCAGCCCAGCATCCTT	TCAGGTGTTCCATCGCATAA
***Tyrp1***	ENSMUSG00000005994	TCTGGCCTCCAGTTACCAAC	GGCTTCATTCTTGGTGCTTC
***Tyrp2***	ENSMUSG00000022129	AACAACCCTTCCACAGATGC	TAGTCACCGGTGGGAAGAAG
***Hprt***	ENSMUSG00000025630	GTTAAGCAGTACAGCCCC	AGGGCATATCCAACAACA

We then hypothesized that RXRs, and in particular RXRα, may directly regulate expression of a subset of genes whose expression was dysregulated in melanocytes lacking *Rxrα/Rxrβ* expression described above. To test that, a region of ∼5 kilobases upstream of the transcriptional start site (TSS) for each gene was interrogated for possible binding motifs of RXRα/NR heterodimers (RXREs) as reported in the TRANSFAC database. Promoter sequences of the predicted target genes were targeted for further investigation and primers were designed to capture those regions (for details see Table 5). Chromatin Immunoprecipitation (ChIP) [20] was performed on nuclear extracts from primary melanocytes using an antibody against RXRα. A mock ChIP was also performed using an IgG antibody. We discovered that the promoter regions of *Cxcl10* and *Fos* had significant enrichment for RXRα binding over the IgG control, as determined by RT-qPCR (Figure 5C, D). Although *in silico* analyses of the promoter region of other dysregulated genes revealed several potential RXREs, no ChIP enrichment at those sites was found (Figure S8). The above results suggest that RXRα directly regulates a small subset of genes involved in skin inflammation and survival; while other dysregulated genes found in the *Rxrα/β^mel−/−^* melanocytes may be regulated either indirectly or *in trans* via DNA binding elements present beyond 5 kb from the TSS.

**Table 5:** List of primer sets used for ChIP-RT-qPCR analysis.

*Gene (distance from TSS)*	Forward Primer (5′-3′)	Reverse Primer (5′-3′)
***Cxcl10 (−3.8 kb)***	TCAGGGCAAGCTTAACAAGG	TGGGATTTGAACTCCTGACC
***Cxcl10 (−3.3 kb)***	GGTTTGTGGTTTGAGGAGGA	GGCCAGGTGAGGATTTCATA
***Cxcl10 (−0.1 kb)***	TCCAAGTTCATGGGTCACAA	GAGTTTCCCTCCCTGAGTCC
***Cxcl10 (3′ UTR)***	CCAACGTGTGAACAAGGAGA	GCGTGCAGTGAGTTGAGGTA
***Fos (−3.2 kb)***	ACACCCGGCATCATTTCTT	CGCCTGGGTAAACAACACAT
***Fos (−1.3 kb)***	GACCCTCAGAATGGAGACGA	AAGAGGTCAGGTGCTTCAGTG
***Fos (3′ UTR)***	TGCAAGGATGTGCTTTTCTG	TCTGGTTTGCAGTGTGGAAG
***Cxcl12 (−4.3 kb)***	GGATGCCCAAGTCCTACAGA	GTTGCTGTCCCTTGAACCAT
***Cxcl12 (−1.4 kb)***	CAGGCATCAGGTATCCCAAG	TCCATTTCTACCGGCTTTTG
***Cxcl12 (3′ UTR)***	CCATTCTCTCTGCCCACATT	GCCGTAGGCTGTTTGAGAAG
***Slit2 (−4.1 kb)***	GAAAGCAGAGGCAGGTGAAT	CCATGTTAGGTGGTAGGAATCG
***Slit2 (3′ UTR)***	CTGCATCAGGAAACTGGACA	CCTCCTGAAGGAAATGTTGC
***Cx3Cl1 (−4.2 kb)***	GGAATGAGTGGGAAAGGTGA	CACGGAATCCTCTGGAGAAA
***Cx3Cl1 (−2.3 kb)***	TCTGAGTTCAAGGCCAGCTT	CCCACTTGGGCAGGTTACTA
***Cx3Cl1 (3′ UTR)***	ATAGGGCTAAAACCCCAGGA	GGTTGAAGGCAGGAGGACTA
***Ccl19 (−4.2 kb)***	GCGGATTTCTGAGTTCAAGG	TTAGTCAGGGGCCAGAAATG
***Ccl19 (−3.2 kb)***	CATTCATTGAGGGCTTGGTT	GAGTGGGCTTTGAGGTTTCA
***Ccl19 (−1.5 kb)***	CCACAACCAAGGCAACTTTT	CCTCATCCTAGCAGCCTTCA
***Ccl19 (3′ UTR)***	GCCATCTTCTATTCCGTCCA	CCTCACGGTTCTTTCTTCCA
***Bad (−3.7/3.8 kb)***	AGCGTCTGATTATCCCGATG	GGATACCAGATGCCCACAGT
***Bad (−3.2 kb)***	AAGGTGAGTCCCAGGCAGT	TGGGAACCAGTCTGTTCTGAG
***Bad (3′ UTR)***	GAGCGTGGCTAGACCCTTG	TGACCCAAATAGGAGCAAATG
***Fgf1 (−2.7 kb)***	AATGTGGCAGAAGAGGCTGT	GTTGTCAGGCCCAGTCTTGT
***Fgf1 (−1.7 kb)***	CTAAGCACGGTGGGAGAAAG	GGGCTTGTCAGGTGTCTACC
***Fgf1 (−1.0 kb)***	AAGCGGTTGACTGCTACCTC	TGTCCCTTGTCCTTGGAGAC
***Fgf1 (3′ UTR)***	TAATTGGGGCTGGCTTACAG	ATGTGGCCCTGTTGGAGTAG
***Tnf (−4.4 kb)***	GATGTCCTTTGTTGGGCAGT	CAGGCTTCCAATGCTCTGTA
***Tnf (−3.8 kb)***	GCCAGCCTGGTCTACAGAGT	GTCTTTGGGGGTTCAAGTCA
***Tnf (−0.6 kb)***	CGCAGTCAAGATATGGCAGA	CTTGGAGGAAGTGGCTGAAG
***Tnf (3′ UTR)***	GTGGGTGGATTGGAAAGAGA	CTGAATAAAGTCGGCCTTGC
***Ccl4 (−4.3 kb)***	GACGTGGAAACTTCCCTGAG	GTGCCCACGGTACTTCACTC
***Ccl4 (−2.6 kb)***	GGTAGACGGGAAACAAACCA	AGAGAAGGGGAGGGAAGAGA
***Ccl4 (3′ UTR)***	TTTGCCTCTATGAGGGTGCT	ACTACGCAGAGCTCCCAGAG

## Discussion

**Figure 6:**
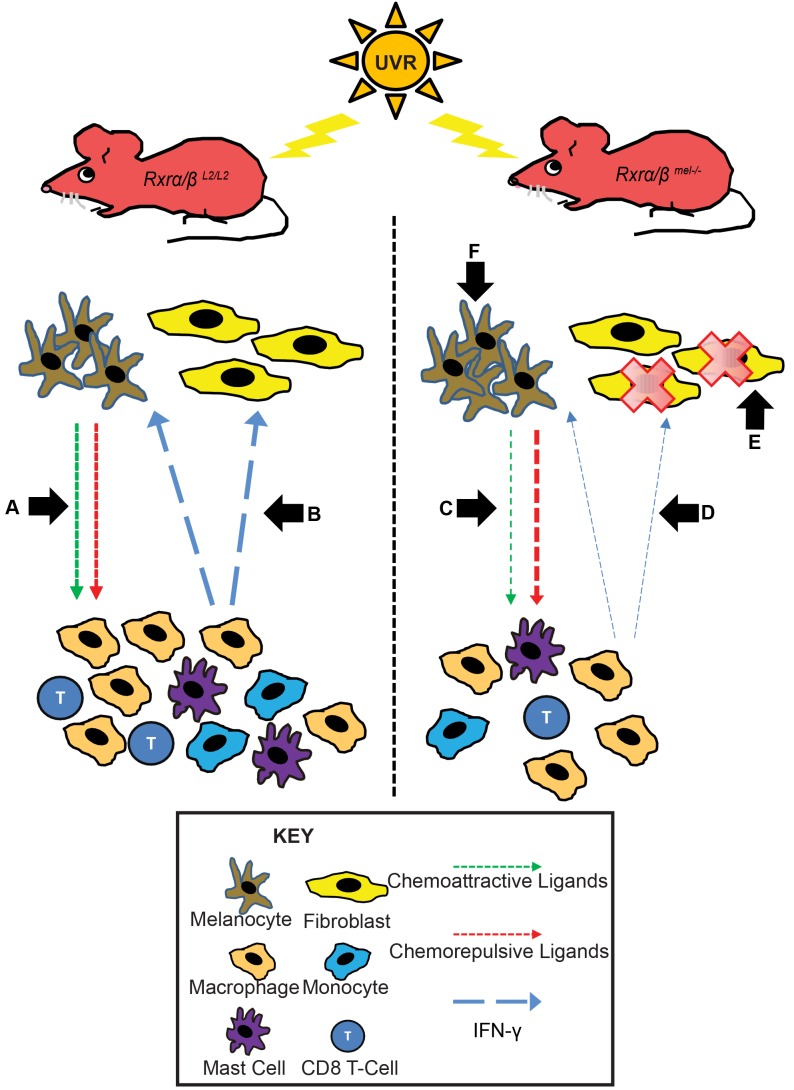
Mechanistic representation of post-UVR defects in *Rxrα/β^mel−/−^* mice compared to control mice. In *Rxrα/β^L2/L2^* control mice post-UVR, melanocytes recruit immune cells via secretion of chemoattractive and repulsive ligands (A). The recruited immune cells secrete IFN-y, which promotes survival of melanocytes/fibroblasts and stimulates additional chemokine secretion from melanocytes via a feedback loop (B). In *Rxrα/β^mel−/−^* mice, there is less secretion of chemoattractive and more secretion of chemorepulsive ligands from the melanocytes (C). As a result, fewer immune cells are recruited as a result of shift in the chemoattractive/repulsive ligands, resulting in reduced available IFN-y (D), negatively influencing fibroblast survival (E). (F) As RXRs α and β are ablated specifically in melanocytes, an endogenous shift in pro- and anti- apoptotic signals results in enhanced survival of melanocytes in *Rxrα/β^mel−/−^* mice relative to controls.

Loss of expression of RXRα has been previously reported during melanoma progression in humans [4], [5]. The present study provides a mechanistic role of RXRs α/β in differentially regulating survival of melanocytes and fibroblasts. We show that RXRs in melanocytes modulate survival of dermal fibroblasts in a non-cell autonomous manner through secretion of paracrine factors and mediate survival of the melanocytes themselves in a cell autonomous manner (Figure 6). Furthermore, our results underscore a novel immunomodulatory role of RXRα/β-mediated nuclear receptor signaling to regulate UVR-induced macrophage activation and cell survival.

RXRα is the predominant RXR isotype expressed in skin [21]; however we also chose to ablate RXRβ due to a compensatory upregulation of *Rxrβ* expression in primary murine melanocytes (Figure S4A). We did not observe a similar compensatory upregulation of *Rxr*α expression following knockdown of *Rxrβ* (Figure S4B). Previously, a functional redundancy was shown to exist between RXRα and RXRβ in epidermal keratinocytes, although RXRα function was clearly dominant [21].

The dermis of the skin is comprised largely of fibroblasts, which maintain the structure of the skin via forming an extracellular matrix comprised of collagen and other ECM proteins. IFN-γ has been previously speculated to have a role in regulating fibroblast apoptosis. Human fibroblasts exposed to gamma radiation exhibited decreased apoptosis 24 and 48 h post-radiation when treated with IFN-γ *in vitro* [22], and IFN-γ was reported to inhibit TRAIL-induced apoptosis in fibroblast-like synovial cells [23]. Indeed, we found that murine dermal fibroblasts express IFNGR1 pre- and post-UVR; and supplementation of the culture medium with IFN-γ following UVR exposure enhances a dose dependent survival of those cells.

The role of interferons in cancer immunosurveillance is complex and at times oppositional. Macrophages recruited after UVR promote survival of melanoma cells, and systemic antibody blockade experiments has established the importance of physiologically relevant IFN-γ in UVR-induced melanocyte activation and melanoma cell survival; underscoring a pro-tumorigenic role of IFN-γ in skin [8]. In contrast, overexpression of SOCS1, a negative regulator of IFN-y mediated signaling, has been previously observed in human melanoma [9], [24]. Neutralization of IFN-γ in a mouse model has been demonstrated to abrogate rejection of transplanted fibrosarcoma cells [10] and increase aggressiveness of MCA-induced tumors arising from these transplanted cells [10]. Furthermore, blockage of the IFN-γ pathway either through knockout of IFNGR1 [9], [11], signal transducer Stat1 [9], [11], or IFN-γ [9], [12] develop higher number of spontaneous or chemical carcinogen-induced tumors; supporting its anti-tumorigenic activity [9]. Altogether, these findings and our data support the notion that melanocytic ablation of RXR α/β may increase susceptibility to intermittent or chronic UVR-induced melanomas. The correlation between fibroblast apoptosis and melanoma susceptibility has never been thoroughly investigated. It is possible that together with enhanced post-UVR melanocyte survival in absence of RXRα/β, the decreased post-UVR survival of fibroblasts we observe may encourage proliferation and propagation of dermal melanomas. It has been found previously that clonal expansion of epidermal cells expressing a mutant p53 is driven by chronic UVB-induced apoptosis of the surrounding cells in the skin [13]; leading to enhanced formation of primary papillomas [13]. Future studies are needed to explore the effects of melanocytic RXR α/β ablation on formation of UVR-induced tumors in adult mice.

Our observation that melanocytic ablation of RXR α/β results in decreased infiltration of F4/80+ macrophages, CD11B+ monocytes, CD8+ T-cells and mast cells suggests defects in expression of paracrine chemokine factors secreted by the melanocytes to mediate immune cell infiltration. Secretion of CCR2 ligands CCL2 and CCL8 from melanocytes following UVR irradiation activates F4/80+ macrophages and enhances their recruitment [8]. Although we did not observe changes in *Ccl2* or *Ccl8* expression after *in vitro* knock-down of *Rxrα/β* in melanocytes, we identified dysregulated post-UVR expression of several genes involved in chemoattraction/chemorepulsion of immune cells. In particular, blockage of chemoattractant CX3CL1 has been previously reported to be reduce macrophage infiltration in experimental autoimmune myositis (EAM) mice [25], indicating that its downregulation in our model could contribute to the reduced macrophage infiltration we observe. Similarly, CCL19 has been reported to be a chemoattractant for several different immune cell types, including lymphocytes [26]–[28], dendritic cells [28], [29], natural killer cells [28], [30], and macrophage progenitors [28], [31]. CCL4 is also reported to be a chemoattractant of macrophages, monocytes, dendritic cells, natural killer cells, and eosinophils [32]. Likewise, *Ccrl2^−/−^* mice have been reported to have a reduced inflammatory response in lung tissue following OVA-induced airway inflammation [33].

Regarding chemorepulsive factors, *Slit2* has a role in regulating axon guidance and neuronal migration through chemorepulsion [34]. SLIT2 has been found to inhibit migration of Langerhans cells [34], leukocytes [35], neutrophils [36], and most interestingly, *Slit2^−/−^* mice were shown to have increased numbers of F4/80+ macrophages [37], underscoring its chemorepulsive properties. Expression of *Slit2* in skin has been reported to be upregulated at both 4 hours and 48 hours following hapten sensitization [34], suggesting *Slit2* can have a role both early and late in an inflammatory response. Likewise, CXCL10 has been shown to repulse plasmacytoid dendritic cells [38]; interestingly CXCL10 has also been shown to inhibit angiogenesis [39]–[41] and overexpression or mutation of CXCL10 in melanoma cells results in slower growth of xenografts [41], [42]. Our results suggest recruitment of RXRα protein on the proximal promoter region of *Cxcl10*, mRNA expression of which is upregulated in our model. Based on our data, CXCL10 may have a significant role in RXR-mediated immune cell recruitment by melanocytes via direct transcriptional regulation.

We did not observe evidence of RXR recruitment on the promoter regions of several genes dysregulated in our model (Figure S8). These results can be reconciled by the fact that expression of these genes may be regulated via alternative mechanisms such as (1) by downstream effectors of RXR, (2) by RXR binding to distal promoter/enhancer regions, and/or (3) regulated *in trans* via enhancer RNAs (eRNAs) [43].

While melanocyte-specific ablation of RXRα/β results in a paracrine effect, altering chemotaxis of immune cells which ultimately affect survival of the dermal fibroblasts, the melanocytes themselves were able to overcome this effect and have enhanced post-UVR survival over controls despite reduced presence of IFN-γ in the microenvironment that modulates UVR-induced melanocyte survival just as in fibroblasts. Our studies utilizing RXR agonist and antagonist suggested that RXRs can independently mediate apoptosis in melanocytes post-UVR via multiple pathways, by transcriptional activation and/or repression of genes. RT-qPCR analyses of pro- and anti-apoptotic genes in isolated *Rxrα/β* double knockdown melanocytes revealed altered expression of several anti-apoptotic and pro-apoptotic genes. In particular, pro-apoptotic genes *Bad* and *Trp53* and anti-apoptotic gene *Fgf1* were found to be dysregulated in a similar fashion in both UVR-exposed *Rxrα/β* double knockdown melanocytes *in vitro* and in FACS-sorted CD117+/CD45− cells from UVR-exposed *Rxrα/β^mel−/−^* skin *in vivo*. Our results suggest that RXRα binds directly to the promoter of *Fos*, the product of which (c-FOS) makes up a subunit of the AP1 transcription factor and when activated can induce apoptosis [44]. Interestingly, *Fos* was found to be downregulated in the *Rxrα/β* double knockdown melanocytes but upregulated in the *in vivo* FACS-sorted cells. However, these two models are not fully homologous as there is cell-cell signaling at play *in vivo*; and different parameters with regard to UVR-dose and time points are required to elicit similar responses in a homogenous cell monolayer versus a multicellular organ. This could well be the case for why some other genes are dysregulated differently in *Rxrα/β* double knockdown melanocytes than in the *in vivo* CD117+/CD45− cells. As our results suggest the promoter of *Fos* is bound by RXRα, it is likely that it is indeed a player in regulating melanocyte apoptosis post-UVR, but in a context-dependent manner. We believe that a shift in homeostasis of pro- and anti-apoptotic signals within the melanocytes themselves in absence of the RXRs might contribute to overcome the negative effects of reduced IFN-γ on cell survival seen in the fibroblasts.

Altogether, ablation of RXRα/β expression in melanocytes appears to have two distinct independent effects induced by UVR: (1) A paracrine immunomodulatory effect reducing infiltrating immune cells and hence survival of dermal fibroblasts owing to reduced available IFN-γ in the microenvironment; and, (2) an independent cell-autonomous effect on survival of the melanocytes themselves due to alteration in the expression level of pro- and anti-apoptotic genes (shift in apoptotic signals) selectively in those cells, allowing them to overcome the negative-effects of reduced IFN-γ on cell survival. Whether these changes enhance susceptibility to melanoma and other types of skin cancer in the long-term, need to be further examined in future using chronic or intermittent UV exposure of the *Rxrα/β^mel−/−^* mice.

## Materials and Methods

### Mice

Generation of *Rxrα/β^L2/L2^* mice has previously been described [45]. To selectively ablate RXRα and RXRβ in melanocytes, mice carrying LoxP-site-containing (floxed) *Rxrα* and *Rxrβ* alleles were bred with hemizygous Tyr-Cre transgenic mice [14] to produce *Rxrα/β^mel−/−^* mice in Mendelian ratios, following Cre-mediated recombination selectively in melanocytes. A semi-quantitative PCR was performed in an Eppendorf thermal cycler using primers to amplify the Cre, L2 and L- RXR alleles [21], [45] as described. Mice were housed in our approved University Animal Facility with 12 h light cycles, food and water were provided ad libitum, and institutional approval was granted for all animal experiments by the Institutional Animal Care and Users Committee (IACUC).

### UVR treatment of mice

Neonatal mice (P4 for data in Figure S7, P2 for all other experiments) were exposed to a single dose of 800 mJ/cm^2^ of UV-B light from a bank of four Philips FS-40 UV sunlamps as described [6]. The irradiance of the sunlamps was measured with an IL-1400A radiometer with an SEE240 UVB detector (International Light). Mice were euthanized 24, 48, 72 and 96 h after UVR and skin samples retrieved. 0 h samples were taken from P2 mice not exposed to UVR.

### Histological analyses

All analyses were performed on 5 µm formalin-fixed paraffin sections. Prior to all procedures, sections were deparaffinized in xylene and rehydrated using graded alcohols. Sections were stained with hematoxylin and eosin (H&E) as previously described [46]. Fontana-Masson staining was performed according to manufacturer's instructions (American MasterTech). Toluidine Blue staining was performed by immersing slides in a solution of 0.1% Toluidine Blue in 1% Sodium Chloride, pH 2.3 for 2 minutes. Slides were then washed/dehydrated by dipping 10 times in 95% EtOH, followed by 10 dips each in 2 changes of 100% EtOH. Slides were cleared in xylene and mounted in DPX mounting medium.

### Immunohistochemistry

For immunohistochemistry (IHC) staining studies, paraffin sections from mouse skin (5 µm thick) were deparaffinized in xylene and rehydrated through graded alcohols. Antigen retrieval was performed in a hot water bath (95°C–100°C) using citrate buffer (pH 6.0) for 20 minutes. Frozen sections from mouse skin (8 µm thick) were post-fixed in cold acetone at −20°C for 10 minutes then air dried at room temperature for 20 minutes. All sections were then washed three times with 0.05% PBS-Tween (PBST); then nonspecific antibody binding was blocked using 10% Normal Goat Serum in PBST at room temperature for 30 minutes. Sections were then incubated overnight at 4°C with primary antibody. Primary antibody incubation was followed by three washes with PBST before addition of the secondary antibodies, which were incubated on the sections for 2 hours at room temperature. Nuclei were counterstained with DAPI (200 ng/mL) for 10 minutes at room temperature. Finally, sections were rinsed with PBST, dehydrated through sequential alcohol washes and then cleared in xylene. Slides were mounted with DPX mounting medium. Antibodies used in IHC are detailed in Table 2. For dual TUNEL-IHC staining, the DeadEnd TUNEL System (Promega) was combined with the above protocol. Sections stained without primary antibody was used as a negative control, and all experiments were performed in triplicates.

### Imaging and quantitation of histological and IHC experiments

Brightfield images were captured with a Leica DME light microscope using the Leica Application Suite software, version 3.3.1. Fluorescent images were captured using a Zeiss AXIO Imager.Z1 with a digital AxioCam HRm and processed using AxioVision 4.8 and Adobe Photoshop. Epidermal thickness was measured using the Leica Application Suite, taking random measurements across 20 image fields per animal; which were then averaged to calculate an average value per animal. Quantifications of cell labeling were performed by randomly choosing multiple fields imaged from several replicate animals in each group and counting cells using ImageJ software (NIH). All slides were analyzed independently in a double-blinded manner by two investigators and significance was determined using a Student's two-tailed t-test as calculated by GraphPad Prism software.

### Primary melanocyte culture

Primary C57BL/6 murine melanocytes were obtained from the Yale University Cell Culture Core. Cells were maintained in a complete growth medium consisting of F-12 nutrient mixture (Ham), 8% FBS, bovine pituitary extract (25 µg/mL), TPA (10 ng/mL), 3-isobutyl-1-methylxanthine (22 µg/mL) and 1× antibiotic/antimycotic. Melanocytes were starved into a quiescent state using a minimal culture medium containing F-12 nutrient mixture (Ham), 8% FBS and 1× antibiotic/antimycotic for 48–72 h prior to all experiments. All cell culture/assays were performed at 37°C, 5% CO_2_.

### Primary fibroblast culture

Primary skin fibroblasts were cultured by removing dorsal and ventral skin from newborn mouse pups and incubating in growth medium containing 5 mg/mL dispase at 4°C overnight with rocking. The next day, the skins were washed in sterile PBS and the epidermis separated and discarded. The dermis was incubated in TrypLE Express (Invitrogen) for 30 minutes at 37°C. The dermis was shredded using forceps, suspended in growth medium (F-12 nutrient mixture (Ham), 8% FBS and 1× antibiotic/antimycotic), and vortexed for 2–3 minutes to shed individual cells. The cell suspension was centrifuged (300× *g*, 3 minutes) and the pellet resuspended in fresh growth medium and plated. Medium was changed the day after plating, and cells were split at a 1∶5 ratio once confluency was reached. Cells were maintained for several passages by culturing in aforementioned growth medium and using a 1∶5 split ratio. All cell culture/assays were performed at 37°C, 5% CO_2_.

### Transfection and sorting of primary melanocytes with shRNA plasmids

Primary melanocytes cultured in complete growth medium (detailed above) were transfected with a 1∶1 mixture of *Rxrα* and *Rxrβ* shRNA-containing plasmids (OriGene, pGFP-V-RS for *Rxrα*, pRFP-C-VS for *Rxrβ*) using the Neon Transfection System (Invitrogen) according to manufacturer's instructions. shRNA targeting sequences are detailed in Table 3. Cells were incubated post-transfection for 2 days, then detached from plates using TrypLE Express (Invitrogen) and centrifuged (300*× g*, 3 minutes). Cell pellets were resuspended in minimal growth medium (detailed above) and placed on ice. Fluorescence-activated cell sorting (FACS) of GFP-positive, RFP-positive, or GFP/RFP double-positive cells was accomplished using a Beckman Coulter MoFlo XDP high-speed cell sorter. Cells were sorted into collection tubes containing a high-serum sorting medium (F-12 nutrient mixture (Ham), 16% FBS and 2× antibiotic/antimycotic) to maximize survival of sorted cells. For UVR studies, sorted cells were re-plated, and medium changed for fresh minimal growth medium once cells had fully adhered (3–4 hours post-plating). Cells were incubated in minimal growth medium for 48 hours prior to use for sample collections or experiments.

### FACS sorting of CD117+/CD45− melanocytes from neonatal mouse skin

P4 neonatal mice were irradiated with UVR as described above. 96 hours post-UVR, mice were sacrificed and dorsal skin was collected. Skin was placed in growth medium [F-12 nutrient mixture (Ham)] containing 5 mg/ml dispase and incubated overnight at 4°C. Skin was then diced using forceps and incubated in TrypLE Select (Invitrogen) for 30 mins at 37°C. Individual cells were shed by physical agitation and suspended in growth medium [(F-12 nutrient mixture (Ham)), 8% FBS]. Cell suspensions were filtered (50 µm), pelleted by centrifugation (300× *g*, 3 minutes) and resuspended in labeling buffer (PBS+2% FBS). Non-specific cell-surface binding was blocked by adding Fc Block (BD Pharmingen) at a concentration of 1 µg/10^6^ cells and incubating on ice for 10 minutes [19]. PE-CD117 and FITC-CD45 antibodies were added at a concentration of 0.2 µg/10^6^ cells [19] and incubated at 4°C for 1 hour with rocking. Two-color FACS sorting was accomplished using a Beckman Coulter MoFlo XDP high-speed cell sorter. Non-labeled and single-labeled samples were used to calibrate the sorter. Cells were sorted directly into Trizol reagent (Invitrogen) for RNA isolation.

### UVR treatment of cells *in vitro*

Prior to UVR, growth medium was removed from culture dishes by aspiration. Cells were washed briefly with sterile PBS, which was then removed by aspiration. Lids from culture dishes were removed, and cell monolayers were exposed a single dose of 10 mJ/cm^2^ of UV-B light from a bank of four Philips FS-40 UV sunlamps as described above. Immediately after UVR, fresh growth medium was added back to the cells and were returned to the growth incubator.

### Immunoblotting analyses

Protein lysates were obtained by collecting cells in a lysis buffer (20 mM HEPES, 250 mM NaCl, 2 mM EDTA, 1% SDS, 10% glycerol, 50 mM NaF, 0.1 mM hemin chloride, 5 mM NEM, 1 mM PMSF and 10 mg/mL leupeptin and aproptinin) [6], [47], [48] followed by sonication. Protein concentration was performed using the BCA assay (Thermo Scientific). Equal amounts of protein extract (9–15 µg depending on experiment) from each lysate were resolved using sodium dodecyl sulfate polyacrylamide gel electrophoresis and transferred onto a nitrocellulose membrane as described. The blots were blocked overnight with 5% nonfat dry milk and incubated with specific antibodies. The antibodies used are detailed in Table 2. After incubation with the appropriate secondary antibody, signals were detected using immunochemiluminescent reagents (GE Healthcare, Piscataway, NJ). Equal protein loading in each lane was confirmed with a β-actin antibody (#A300-491, Bethyl).

### ELISA assays

Protein lysates were prepared as described above. Assays were performed using the eBioscience ‘Mouse IFNg ‘Femto-HS’ High Sensitivity ELISA Ready-Set-Go!’ kit according to manufacturer's protocol. 25 µg of protein was loaded per well. Lysates from three mice per group were used and assays performed in triplicate wells. Student's two-tailed t-test was performed using GraphPad Prism software.

### Annexin V/Propidium Iodide assay of apoptosis in cultured cells

Cultured primary melanocytes or fibroblasts were irradiated with UVR as described above. Following UVR, fresh growth medium was added to cells supplemented with treatment, such as 1 µM BMS-649 (A gift from Hinrich Gronemeyer; IGBMC, France) or 100 nM HX-531 (Tocris Bioscience) (Figure 2G); recombinant IFN-y (Millipore) (Figure 3E), or appropriate vehicles. Additionally, cells treated with BMS-649 or HX-531 were pre-treated for 24 h prior to UVR. 24 h post-UVR, cells were harvested and assayed for apoptosis using an Annexin V/Propidium Iodide assay. The assay was performed using a Tali Image-Based Cytometer (Invitrogen) and Tali Apoptosis Kit (Invitrogen) according to manufacturer's protocol. 9 or 20 Tali image fields for each sample were analyzed depending on experiment. All assays were performed in triplicate. Student's two-tailed t-test was performed using GraphPad Prism software.

### Reverse transcription–quantitative PCR (RT-qPCR) analyses of transcriptional changes

Total RNA was extracted from cell monolayers using Trizol (Invitrogen), followed by purification using RNEasy spin columns (Qiagen). cDNA was synthesized using SuperScript III RT (Invitrogen). Amplification was performed on an ABI Real Time PCR machine using a QuantiTect SYBR Green PCR kit (Qiagen), and all targets were normalized to the housekeeping gene *Hprt*. All reactions were performed in quadruplicates or sextuplicates. Melting curve analyses were performed to ensure specificity of amplification. Student's two-tailed t-test was performed using GraphPad Prism software.

### *In silico* discovery of RXREs and chromatin immunoprecipitation RT-qPCR (ChIP-RT-qPCR) analyses

*In silico* discovery of consensus RXR response elements (RXREs) was performed on a 5 kb region upstream from the transcription start site, using the Fuzznuc utility (EMBOSS). Primers were designed to capture regions containing potential RXREs. Cultured primary melanocytes were crosslinked using formaldehyde. 3×10^6^ cells were used for each ChIP. Chromatin was sheared using 10 sonication cycles of 10 s with 20% amplitude. ChIP was performed using either 2 µg of an RXRα antibody (Santa Cruz Biotechnology, see Table 2) or non-specific IgG (Santa Cruz Biotechnology). RT-qPCR amplification was performed on an ABI Real Time PCR machine using a QuantiTect SYBR Green PCR kit (Qiagen). All reactions were performed in quadruplicates, and average Ct values were used for calculating percent input. Melting curve analyses were performed to ensure specificity of amplification. Each assay was performed in replicate three times. Student's two-tailed t-test was performed using GraphPad Prism software.

## Supporting Information

Figure S1 No phenotypic changes observed in adult *Rxrα/β^mel−/−^* mice compared to controls; post-UVR epidermal thickness and melanocyte numbers are unchanged following ablation of RXR α and β in melanocytes. (A) Comparison of adult *Rxrα/β^mel−/−^* to their *Rxrα/β^L2/L2^* controls. No phenotypic differences or effects on viability are observed. (B, C) H&E staining of skin sections following a single dose of UVR. At least five individual measurements of epidermal thickness were made on 20 different fields. (D, E) Fontana-Masson staining of skin sections following a single dose of UVR. Black staining indicates melanin. Nuclei were counter-stained with Nuclear Fast Red. Black-stained cells were presumed to be melanocytes and quantitated. Yellow arrows indicate epidermal extrafollicular melanocytes; green arrows indicate extrafollicular dermal melanocytes. For all images: E = epidermis, D = dermis, HF = Hair Follicle. Scale bar = 50 µm.
(TIF)

Figure S2 Levels of post-UVR cyclopyrimidine dimer (CPD) formation and oxidative DNA damage (8-oxo-dG) across all skin compartments is unchanged as a result of ablating RXR α and β from melanocytes. IHC of skin sections following a single dose of UVR. (A, B) CPD+ cells are indicated by red staining. CPD formation in melanocytes was assessed by co-labeling for the melanocyte-specific protein TYRP1, indicated by green staining. DAPI (blue) was used to counter-stain nuclei. (C, D) 8-oxo-dG+ cells are indicated by red staining. CPD formation in melanocytes was assessed by co-labeling for TYRP1, indicated by green staining. DAPI (blue) was used to counterstain nuclei for all images. E = epidermis, D = dermis, HF = hair follicle. Scale bars = 50 µm.
(TIF)

Figure S3 Loss of melanocytic RXRs α and β also alters profile of infiltrating immune cells other than macrophage following UV radiation. (A–D) Histological characterizations of skin sections following a single dose of UVR. (A) Monocytes are labeled by red staining, indicating cells positive for the monocyte marker CD11B by IHC. Yellow arrows designate positive cells; boxes indicate clusters of positive cells. ** = p≤0.01. (B) IHC labeling for CD8-positive T-Cells, as indicated by red immunofluorescence. Yellow arrows designate positive cells. *** = p≤0.001. (C) Toluidine Blue staining of skin sections. Mast cells are indicated by purple staining. ** = p≤0.01. (D) IHC labeling for CD3-positive T-Cells, as indicated by red immunofluorescence. Yellow arrows designate positive cells. DAPI (blue) was used to counterstain nuclei in all fluorescent images. E = epidermis, D = dermis, HF = hair follicle. Scale bars = 50 µm.
(TIF)

Figure S4 Compensatory upregulation of *Rxrβ* expression when *Rxrα* is knocked down in melanocytes. Expression of each *Rxr* mRNA transcript (*α* or *β*) when the other is knocked down using shRNA, measured by RT-qPCR. (A) *Rxrβ* is upregulated when *Rxrα* is knocked down in primary murine melanocytes; (B) there is not a similar compensatory upregulation of *Rxrα* when *Rxrβ* is knocked down. All cells were sorted for shRNA plasmid transfection by FACS, using either a GFP or RFP marker gene. # = No Statistically Significant Difference, *** = p≤0.001, **** = p≤0.0001.
(TIF)

Figure S5 Analysis of peak mRNA expression of chemokines *Ccl2* and *Ccl8* post-UV in cultured wild-type melanocytes. Expression of mRNA transcripts for *Ccl2* and *Ccl8* following UV-B radiation of cultured melanocytes was measured using RT-PCR. (A) *Ccl2* expression was highest 6 hours following treatment of cells with 10 mJ/cm^2^ UV-B. Similarly, expression of *Ccl8* (B) also peaked at the same time point and UVR dose. In both cases lower UVR doses resulted in a reduced response, and all elevated expression was attenuated 24 hours post-UVR.
(TIF)

Figure S6 RT-qPCR arrays to determine altered expression of chemokines/receptors and apoptosis-related genes in RXR knockdown melanocytes post-UVR; and re-validation of results. (A, B) Heat maps generated by RT-qPCR arrays (SA Biosciences) for mouse chemokines (PAMM-022) (A) and mouse cancer (PAMM-033) (B). Heat maps reflect changes in gene expression in UVR-treated primary melanocytes with *Rxr α* and *β* knocked down using shRNA. (C, D) Several genes of interest found to be altered in RXR knockdown melanocytes were verified in biological replicates using our own primer sets. Primers spanning exon junctions were designed independently, and assays were performed on biological replicates of the sample used in the array. # = no statistical significance, ** = p≤0.01, *** = p≤0.001, **** = p≤0.0001. Re-validations of several other genes are shown in Figure 5. (E, F) BioTapestry representation of fold changes as determined by RT-qPCR arrays.
(TIF)

Figure S7 Melanocytes isolated by FACS from UVR-treated *Rxrα/β^mel−/−^* mouse skin show similar gene dysregulation to cultured *Rxrα/β* double shRNA knockdown melanocytes. (A) Live cells were collected from neonatal mouse skin (96 hours post-UVR) and dual-labeled with fluorescent-conjugated antibodies to cell surface antigens CD117 and CD45 in order to isolate CD117+/CD45− melanocytes. (B) The FACS-isolated CD117+/CD45− cells show upregulated mRNA expression of several melanocyte markers compared to CD117+/CD45+ control cells (non-melanocytes), confirming the success of the sort. (C, D) mRNA expression of several chemokines (C) and apoptosis-related genes (D) previously found dysregulated in irradiated cultured *Rxrα/β* double shRNA knockdown melanocytes (Figure 5) showed similar dysregulations in the isolated CD117+/CD45− cells from *Rxrα/β^mel−/−^* mice compared to cells isolated from control mice. In particular, chemokines *Cxcl10, Slit2, Ccl19, Cx3Cl1, Ccl4* and apoptosis-related genes *Fgf1, Bad* and *Trp53* were dysregulated in a similar trend to cultured Rxrα/β double shRNA knockdown melanocytes. ψ = no detectable expression in *Rxrα/β^L2/L2^*, only in *Rxrα/β^mel−/−^*. * = p≤0.05, ** = ≤0.01, *** = p≤0.001.
(TIF)

Figure S8 *In silico* analysis was used to find potential RXR response elements using Fuzznuc motif finder. (A–H) These candidate binding sites were tested for enrichment using ChIP-RT-qPCR. A mock ChIP using a control IgG antibody was also performed. Arrows indicate targeting regions for primers. No significant enrichment was found for these genes. For significantly enriched genes, see Figure 5.
(TIF)
