# Supplementary figures and images for: Retinoid-X-Receptors (α/β) in Melanocytes Modulate Innate Immune Responses and Differentially Regulate Cell Survival following UV Irradiation
Source: PLoS Genet. 2014 May 8;10(5):e1004321. doi: 10.1371/journal.pgen.1004321 (PMC4014444; doi:10.1371/journal.pgen.1004321)

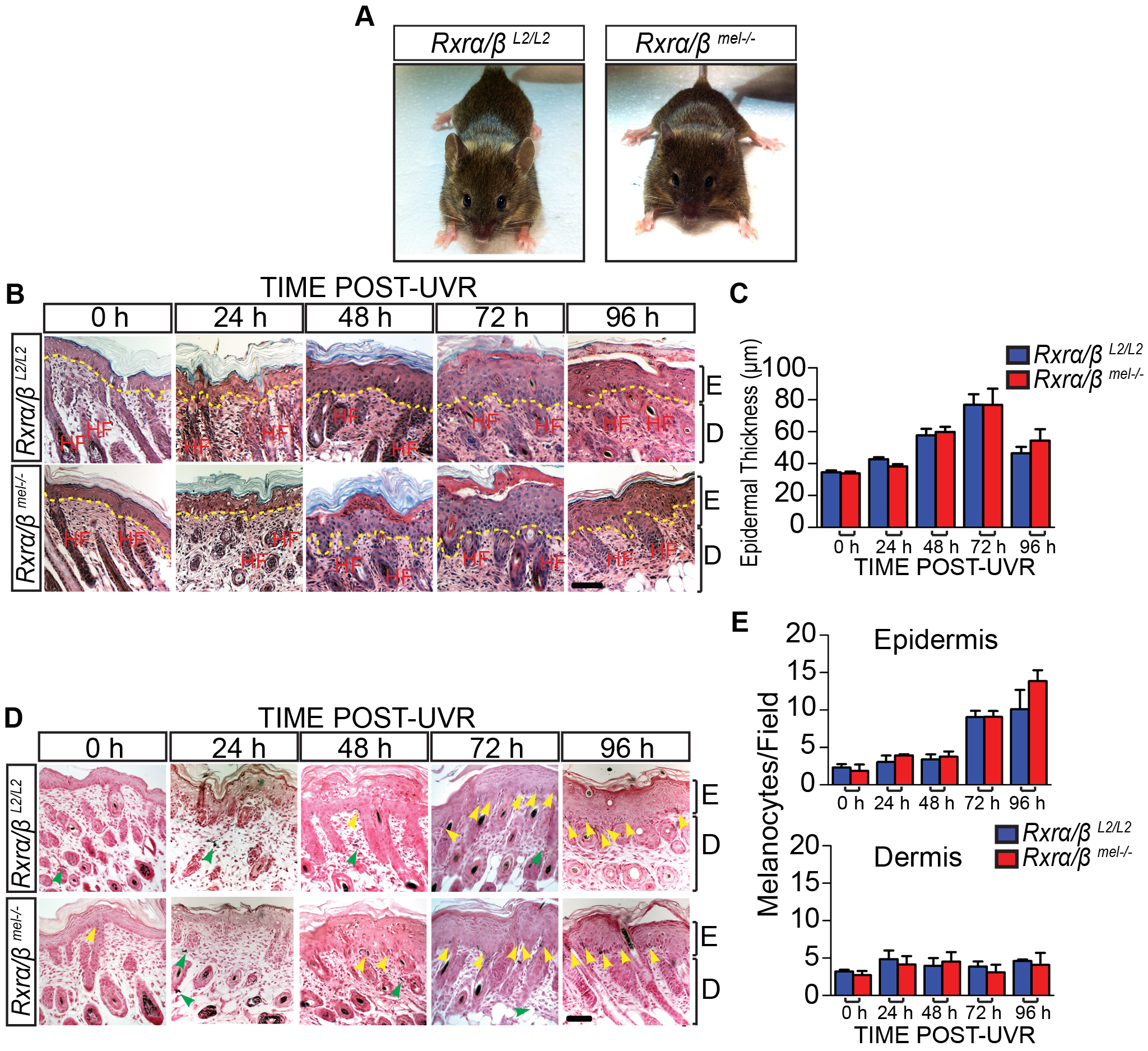

Supplement: Figure S1 — No phenotypic changes observed in adult Rxrα/βmel−/− mice compared to controls; post-UVR epidermal thickness and melanocyte numbers are unchanged following ablation of RXR α and β in melanocytes. (A) Comparison of adult Rxrα/βmel−/− to their Rxrα/βL2/L2 controls. No phenotypic differences or effects on viability are observed. (B, C) H&E staining of skin sections following a single dose of UVR. At least five individual measurements of epidermal thickness were made on 20 different fields. (D, E) Fontana-Masson staining of skin sections following a single dose of UVR. Black staining indicates melanin. Nuclei were counter-stained with Nuclear Fast Red. Black-stained cells were presumed to be melanocytes and quantitated. Yellow arrows indicate epidermal extrafollicular melanocytes; green arrows indicate extrafollicular dermal melanocytes. For all images: E = epidermis, D = dermis, HF = Hair Follicle. Scale bar = 50 µm. (TIF) [file pgen.1004321.s001.tif]

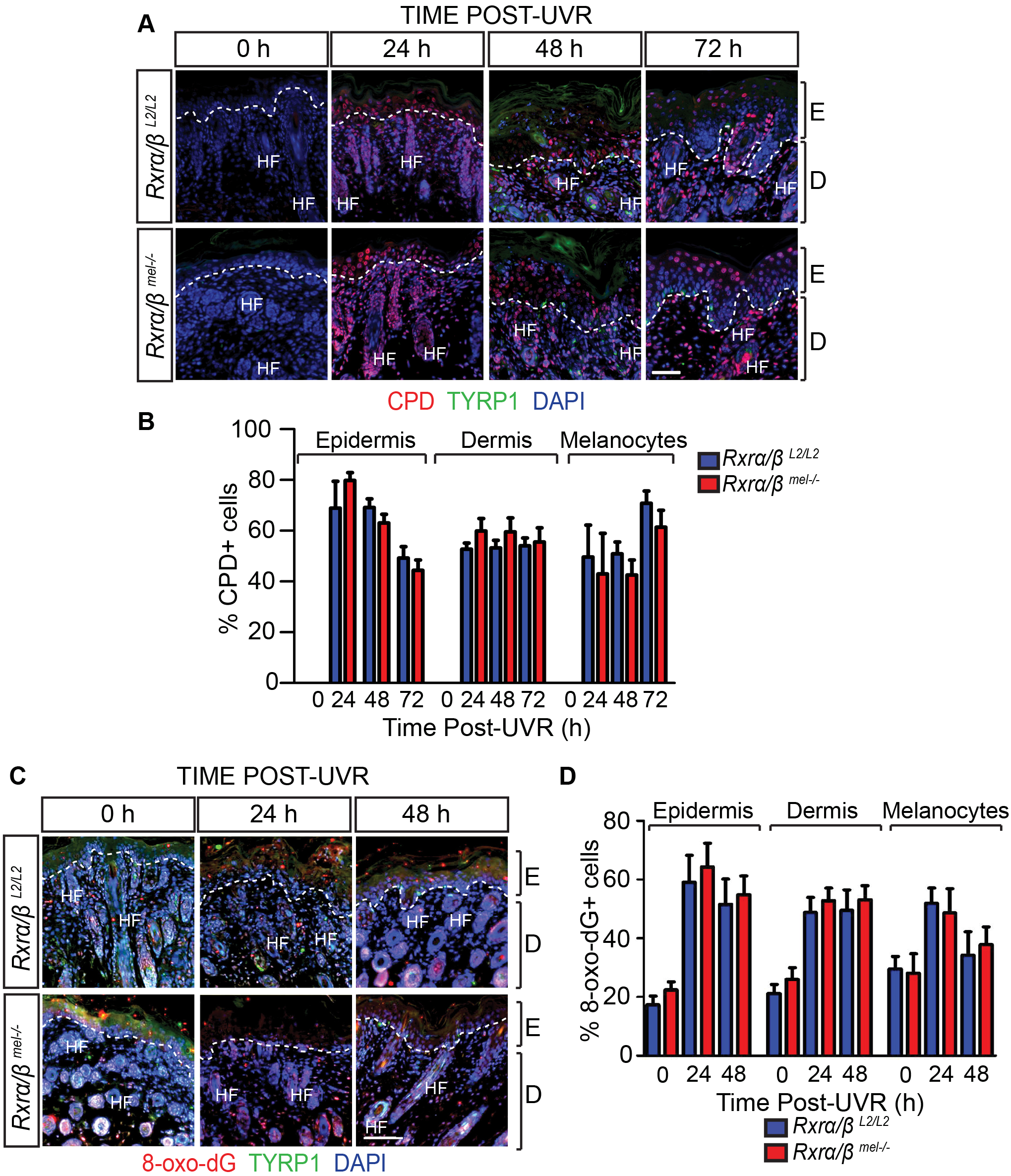

Supplement: Figure S2 — Levels of post-UVR cyclopyrimidine dimer (CPD) formation and oxidative DNA damage (8-oxo-dG) across all skin compartments is unchanged as a result of ablating RXR α and β from melanocytes. IHC of skin sections following a single dose of UVR. (A, B) CPD+ cells are indicated by red staining. CPD formation in melanocytes was assessed by co-labeling for the melanocyte-specific protein TYRP1, indicated by green staining. DAPI (blue) was used to counter-stain nuclei. (C, D) 8-oxo-dG+ cells are indicated by red staining. CPD formation in melanocytes was assessed by co-labeling for TYRP1, indicated by green staining. DAPI (blue) was used to counterstain nuclei for all images. E = epidermis, D = dermis, HF = hair follicle. Scale bars = 50 µm. (TIF) [file pgen.1004321.s002.tif]

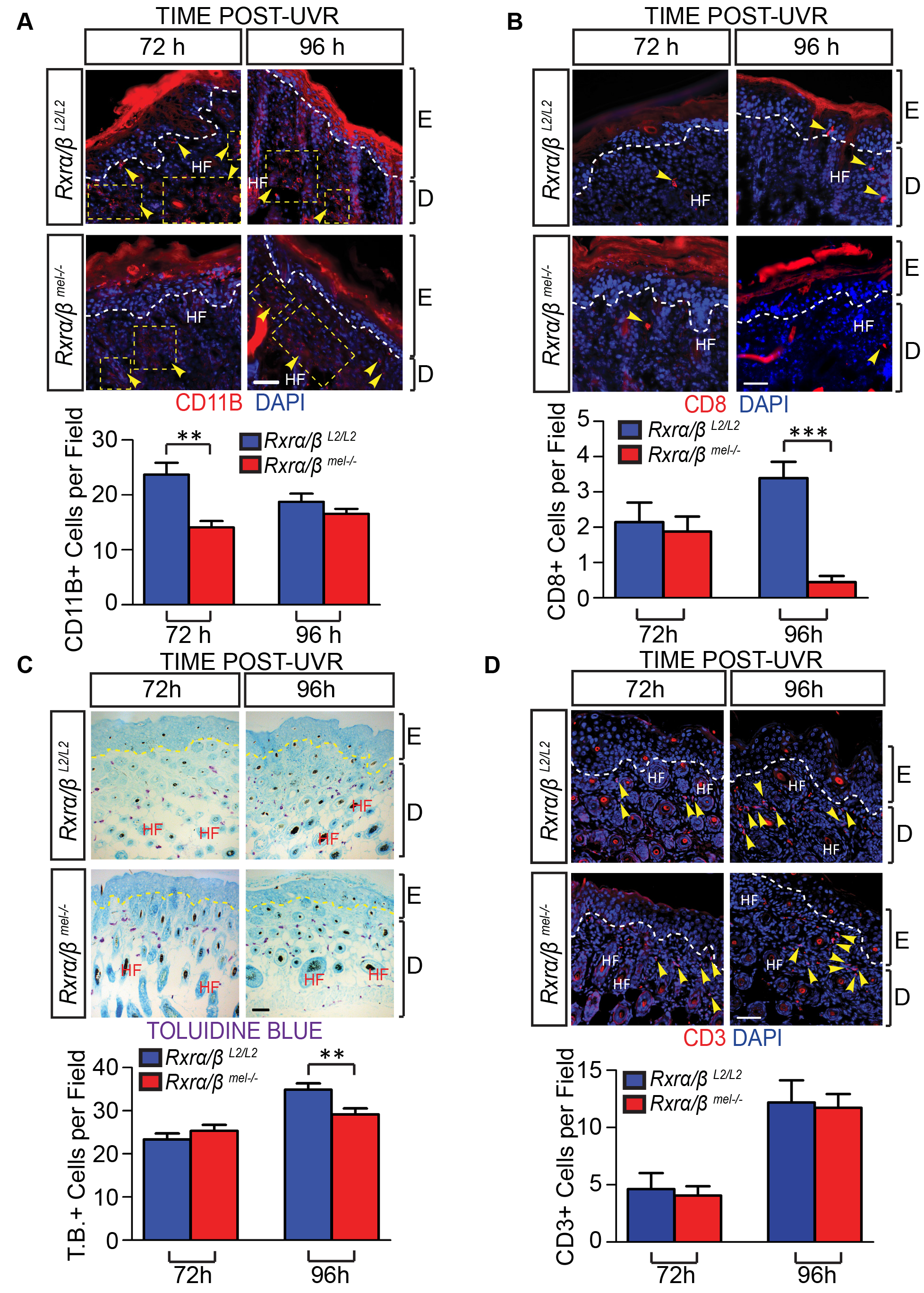

Supplement: Figure S3 — Loss of melanocytic RXRs α and β also alters profile of infiltrating immune cells other than macrophage following UV radiation. (A–D) Histological characterizations of skin sections following a single dose of UVR. (A) Monocytes are labeled by red staining, indicating cells positive for the monocyte marker CD11B by IHC. Yellow arrows designate positive cells; boxes indicate clusters of positive cells. ** = p≤0.01. (B) IHC labeling for CD8-positive T-Cells, as indicated by red immunofluorescence. Yellow arrows designate positive cells. *** = p≤0.001. (C) Toluidine Blue staining of skin sections. Mast cells are indicated by purple staining. ** = p≤0.01. (D) IHC labeling for CD3-positive T-Cells, as indicated by red immunofluorescence. Yellow arrows designate positive cells. DAPI (blue) was used to counterstain nuclei in all fluorescent images. E = epidermis, D = dermis, HF = hair follicle. Scale bars = 50 µm. (TIF) [file pgen.1004321.s003.tif]

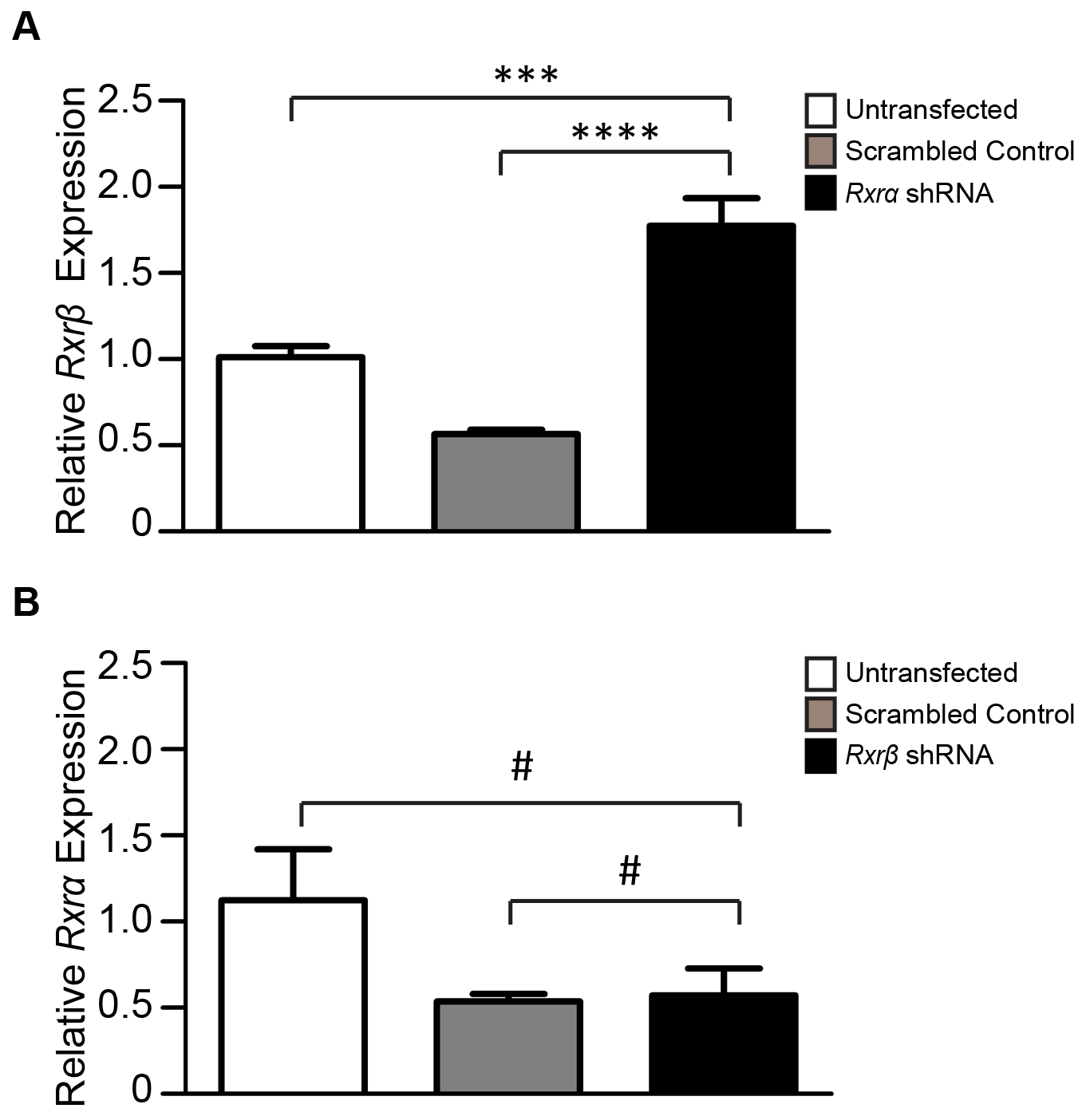

Supplement: Figure S4 — Compensatory upregulation of Rxrβ expression when Rxrα is knocked down in melanocytes. Expression of each Rxr mRNA transcript (α or β) when the other is knocked down using shRNA, measured by RT-qPCR. (A) Rxrβ is upregulated when Rxrα is knocked down in primary murine melanocytes; (B) there is not a similar compensatory upregulation of Rxrα when Rxrβ is knocked down. All cells were sorted for shRNA plasmid transfection by FACS, using either a GFP or RFP marker gene. # = No Statistically Significant Difference, *** = p≤0.001, **** = p≤0.0001. (TIF) [file pgen.1004321.s004.tif]

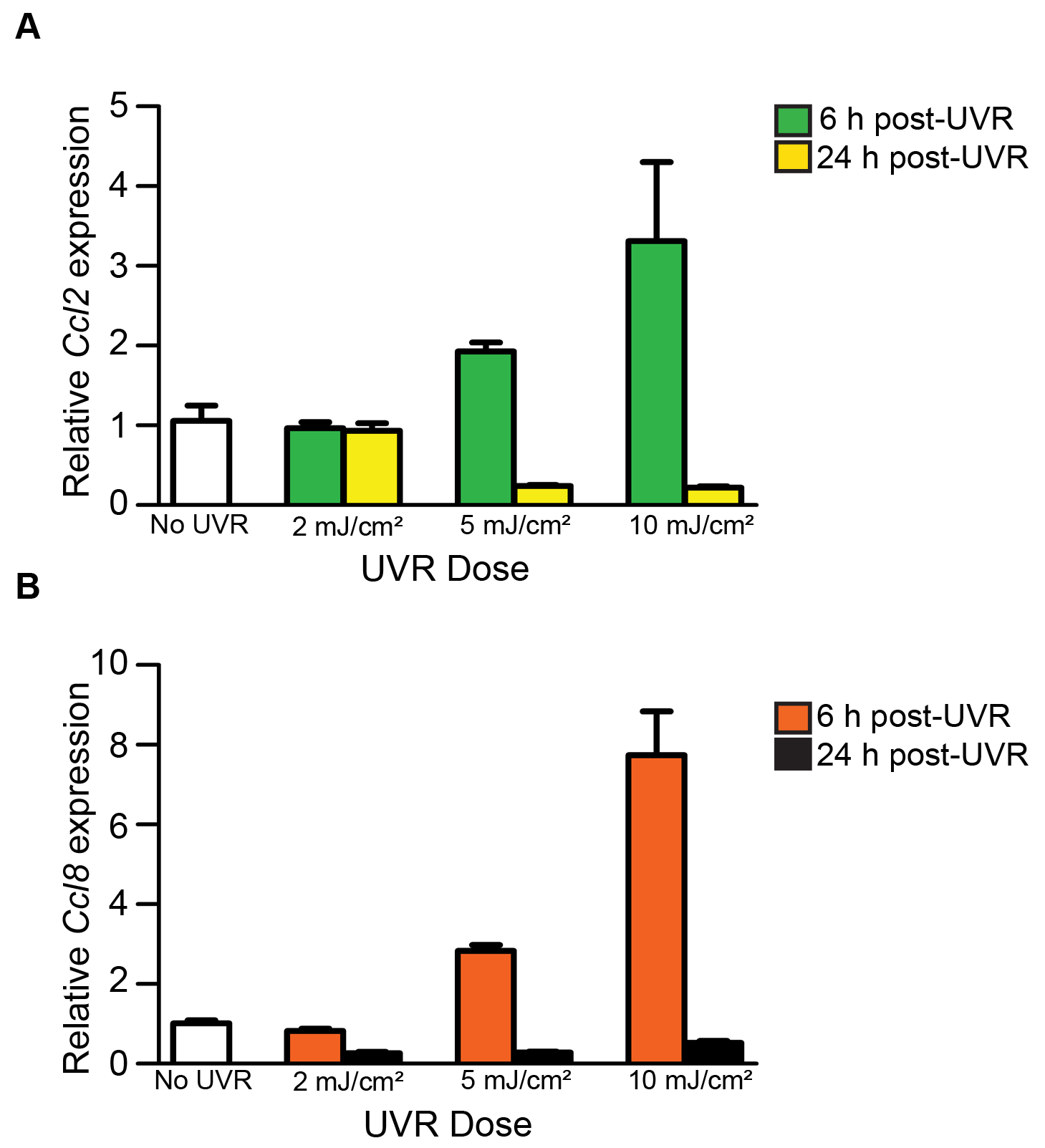

Supplement: Figure S5 — Analysis of peak mRNA expression of chemokines Ccl2 and Ccl8 post-UV in cultured wild-type melanocytes. Expression of mRNA transcripts for Ccl2 and Ccl8 following UV-B radiation of cultured melanocytes was measured using RT-PCR. (A) Ccl2 expression was highest 6 hours following treatment of cells with 10 mJ/cm2 UV-B. Similarly, expression of Ccl8 (B) also peaked at the same time point and UVR dose. In both cases lower UVR doses resulted in a reduced response, and all elevated expression was attenuated 24 hours post-UVR. (TIF) [file pgen.1004321.s005.tif]

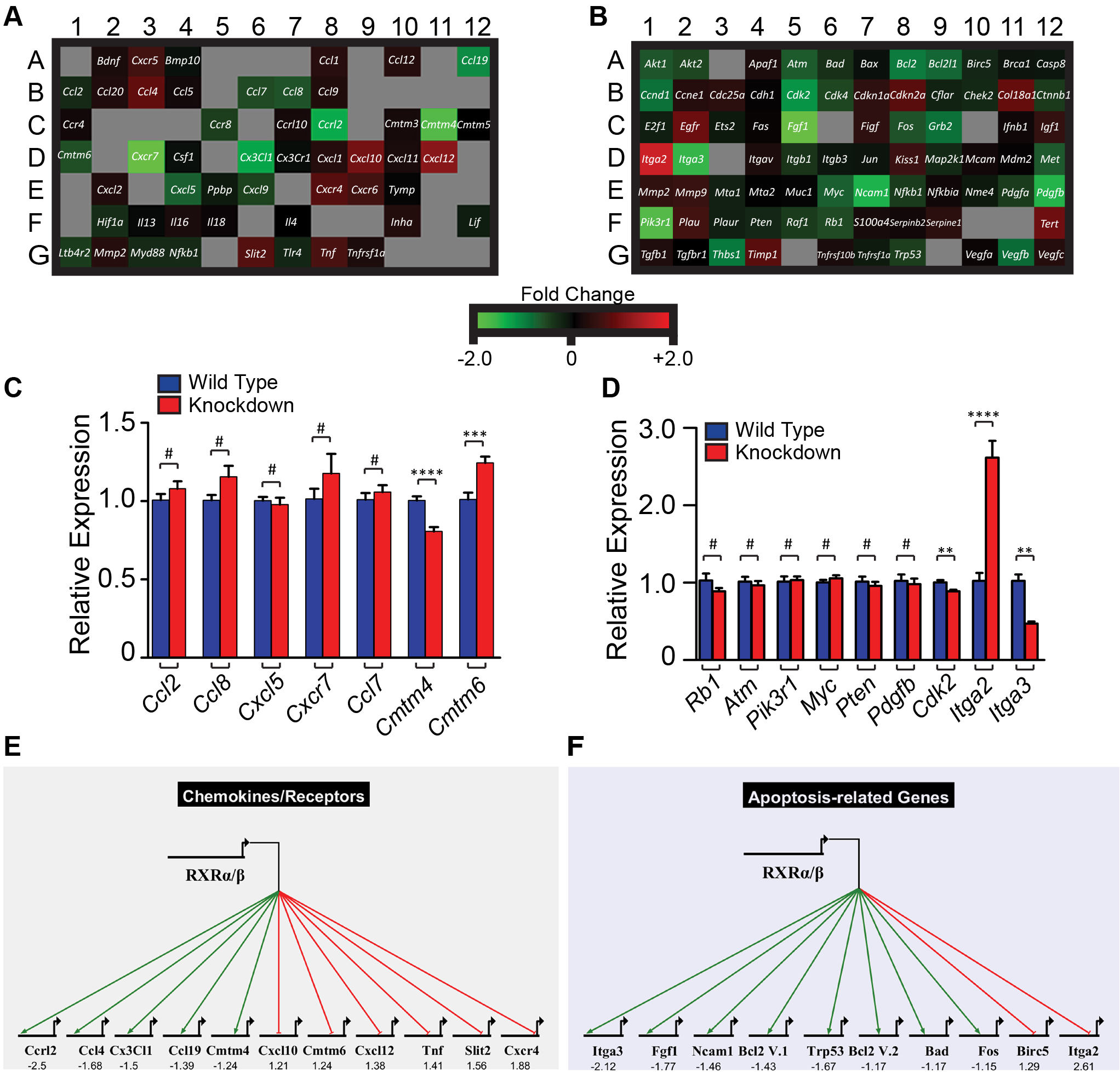

Supplement: Figure S6 — RT-qPCR arrays to determine altered expression of chemokines/receptors and apoptosis-related genes in RXR knockdown melanocytes post-UVR; and re-validation of results. (A, B) Heat maps generated by RT-qPCR arrays (SA Biosciences) for mouse chemokines (PAMM-022) (A) and mouse cancer (PAMM-033) (B). Heat maps reflect changes in gene expression in UVR-treated primary melanocytes with Rxr α and β knocked down using shRNA. (C, D) Several genes of interest found to be altered in RXR knockdown melanocytes were verified in biological replicates using our own primer sets. Primers spanning exon junctions were designed independently, and assays were performed on biological replicates of the sample used in the array. # = no statistical significance, ** = p≤0.01, *** = p≤0.001, **** = p≤0.0001. Re-validations of several other genes are shown in Figure 5. (E, F) BioTapestry representation of fold changes as determined by RT-qPCR arrays. (TIF) [file pgen.1004321.s006.tif]

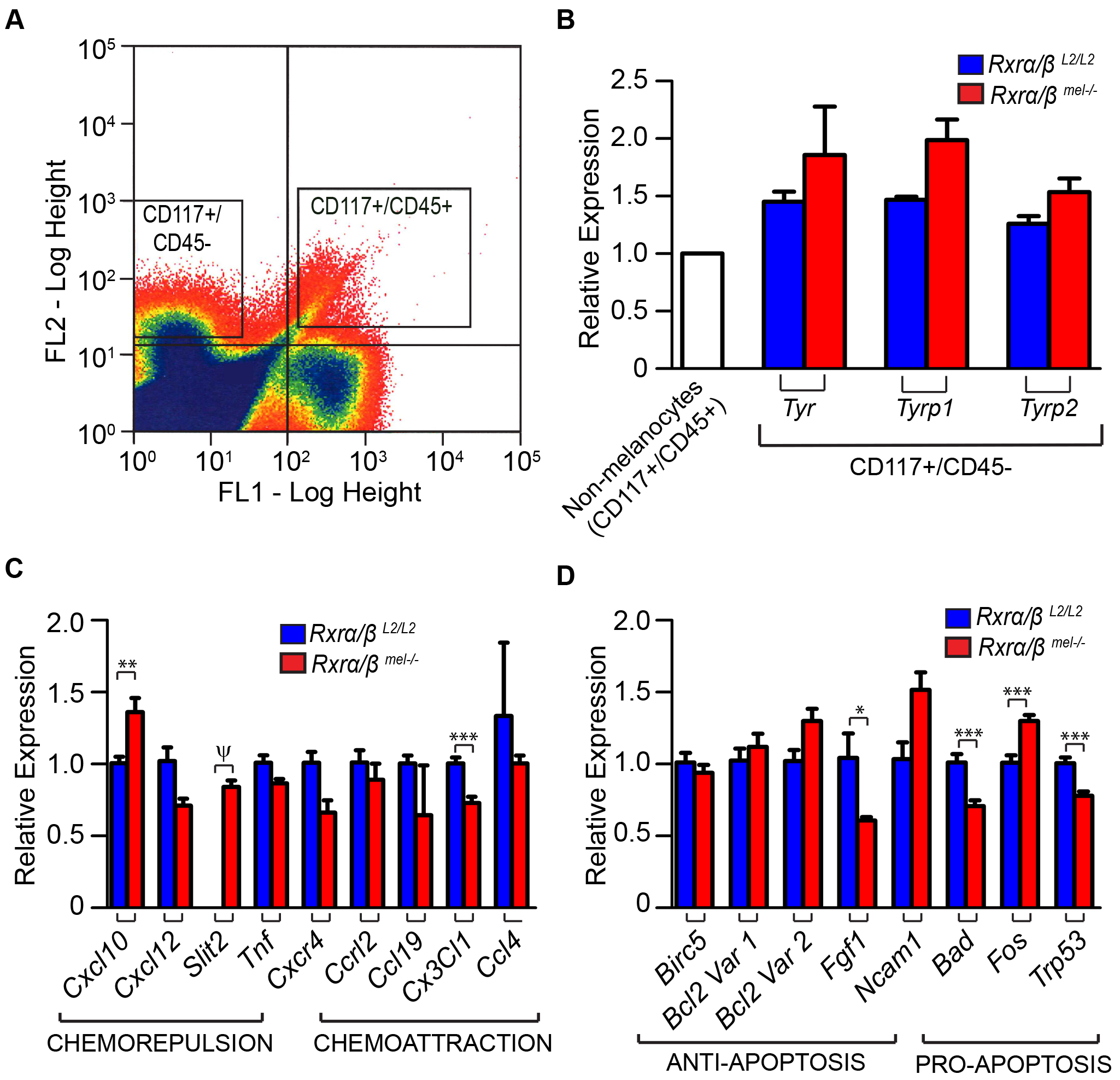

Supplement: Figure S7 — Melanocytes isolated by FACS from UVR-treated Rxrα/βmel−/− mouse skin show similar gene dysregulation to cultured Rxrα/β double shRNA knockdown melanocytes. (A) Live cells were collected from neonatal mouse skin (96 hours post-UVR) and dual-labeled with fluorescent-conjugated antibodies to cell surface antigens CD117 and CD45 in order to isolate CD117+/CD45− melanocytes. (B) The FACS-isolated CD117+/CD45− cells show upregulated mRNA expression of several melanocyte markers compared to CD117+/CD45+ control cells (non-melanocytes), confirming the success of the sort. (C, D) mRNA expression of several chemokines (C) and apoptosis-related genes (D) previously found dysregulated in irradiated cultured Rxrα/β double shRNA knockdown melanocytes (Figure 5) showed similar dysregulations in the isolated CD117+/CD45− cells from Rxrα/βmel−/− mice compared to cells isolated from control mice. In particular, chemokines Cxcl10, Slit2, Ccl19, Cx3Cl1, Ccl4 and apoptosis-related genes Fgf1, Bad and Trp53 were dysregulated in a similar trend to cultured Rxrα/β double shRNA knockdown melanocytes. ψ = no detectable expression in Rxrα/βL2/L2, only in Rxrα/βmel−/−. * = p≤0.05, ** = ≤0.01, *** = p≤0.001. (TIF) [file pgen.1004321.s007.tif]

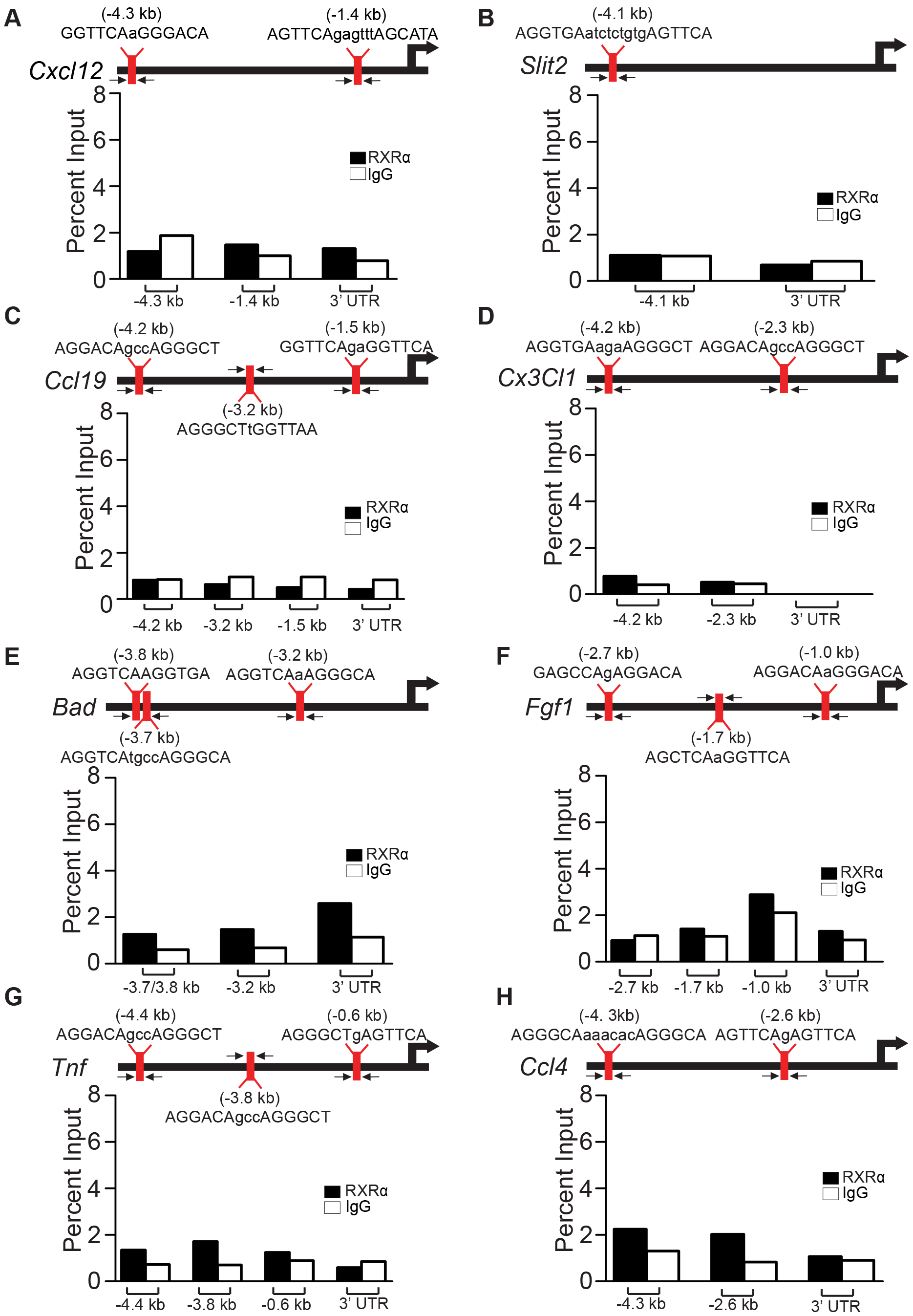

Supplement: Figure S8 — In silico analysis was used to find potential RXR response elements using Fuzznuc motif finder. (A–H) These candidate binding sites were tested for enrichment using ChIP-RT-qPCR. A mock ChIP using a control IgG antibody was also performed. Arrows indicate targeting regions for primers. No significant enrichment was found for these genes. For significantly enriched genes, see Figure 5. (TIF) [file pgen.1004321.s008.tif]
